# TBC1D15 Inhibits Autophagy of Microglia through Maintaining the Damaged Swelling Lysosome in Alzheimer’s Disease

**DOI:** 10.14336/AD.2024.1373

**Published:** 2025-12-13

**Authors:** You Wu, Yong-ming Zhou, Wei Wu, Wan-rong Jiang, Xin-yuan Zhang, Si-yuan Song, Zhao-hui Yao

**Affiliations:** ^1^Geriatrics department, Renmin hospital of Wuhan University, Wuhan 430060, China.; ^2^Department of Clinical Laboratory, Renmin Hospital of Wuhan University, Wuhan 430060, China.; ^3^Department of Neuroscience, Baylor College of Medicine, Houston, Texas, 77030, USA.

**Keywords:** Autophagy, microglia, TBC1D15, Alzheimer’s disease;

## Abstract

Autophagy in microglia is essential for the clearance of amyloid-beta (Aβ) and amyloid plaques in Alzheimer's disease. However, reports regarding the levels of autophagy in microglia have been inconsistent; some studies indicate an early enhancement followed by a subsequent reduction, while others describe a persistently weakened state. Notably, there is a lack of systematic studies documenting the temporal changes in microglial autophagy. TBC1D15, a Rab GTPase, plays a crucial role in lysosomal membrane repair, yet its function in regulating microglial autophagy in Alzheimer's disease remains unexplored. Current research suggests that microglial autophagy is activated in 3-month-old AD mice but gradually decreases by 12 months of age. Furthermore, TBC1D15 levels are significantly elevated in the lysosomes of microglia in Alzheimer's disease. Silencing TBC1D15 markedly inhibits swelling and Aβ phagocytosis in BV2 cells following Aβ treatment while simultaneously promoting autophagy and lysophagy. LIMP II/ATG8-TBC1D15-Dynamin2/RAB7 might participate in lysosome swelling of microglia in AD. These findings indicate that TBC1D15 in microglia is critical for the decline of autophagy in Alzheimer's disease. It is suggested that targeting microglial TBC1D15 may be an important strategy for enhancing autophagy, which facilitates the clearance of amyloid plaques as a therapeutic approach for Alzheimer's disease.

## INTRODUCTION

Autophagy is an important intracellular process that removes metabolic waste, damaged molecules, organelles, and engulfed materials within the cell through the degradation systems of lysosomes [1-3]. Autophagy in microglia of Alzheimer's disease (AD) plays an important role in clear Aβ [4]. Autophagy in microglia decreases in AD, which is closely related to the AD pathological process. The enhanced autophagy in microglia can boost clearing Aβ and reduce the amyloid plaque through multiple pathways, such as PPARA[5], pituitary adenylate cyclase-activating polypeptide (PACAP) [6], TRL4 [7], and microRNAs [8]. Plenty of studies have shown that autophagy in microglia decreases even in the early stage [9]. However, some studies show that the autophagy of microglia was activated, especially for microglia around amyloid plaques, in AD models [4, 9]. It was postulated that autophagy dysfunction in different types of cells and different stages has distinct demonstration [10, 11]. However, how the autophagy in different stages and cell types of AD remains unclear. And the exact mechanisms of autophagy decrease are also elusive.

Autophagy can realize the degradation process through autophagic lysosome. In the AD brain, the damaged lysosome by Aβ aggregation swelled and was filled with plenty of undegraded Aβ aggregation [12]. These swelling lysosomes decrease the autophagy degradation of Aβ in concomitance with high Ph value and decreased cathepsin activity [13]. Due to the fact that in AD, the Aβ aggregates initially phagocytosed by microglia can be promptly degraded in lysosomes through autophagy, significant accumulation in lysosomes is not observed. However, over time, the phagocytosis of Aβ by microglial lysosomes gradually increases, and lysosomal swelling begins to manifest. The dynamic changes in microglial phagocytosis of Aβ and lysosomal swelling damage have not yet been presented in detail. Moreover, it remains unclear how the gradual swelling of lysosomes in microglia occurs in AD—whether it is due to an increasing rate of Aβ phagocytosis surpassing the degradation rate of the lysosomes or whether lysosomal damage occurred early in the process of Aβ degradation by microglia.

Silica, as a non-toxic substance, can be phagocytosed by microglia and enter lysosomes, leading to lysosomal swelling and damage, which induces lysosomal autophagy to clear the damaged lysosomes [14]. Aβ aggregates, as a toxic substance, can damage cell membranes and lysosomal membranes through a carpeting effect, a detergent effect, and ion-channel pore formation in its oligomeric or fibrillary forms [15]. Silica can increase the osmotic pressure of lysosomal crystals within lysosomes, causing lysosomal swelling and increased lysosomal membrane permeability, thereby damaging the lysosomes. Similarly, Aβ aggregates may lead to elevated colloidal osmotic pressure within lysosomes due to delayed degradation by proteases, resulting in lysosomal swelling and increased lysosomal membrane permeability, ultimately causing lysosomal damage. However, the key processes and molecular mechanisms underlying lysosomal damage and repair induced by Aβ aggregates remain unclear.

TBC (Tre2/Bub2/Cdc16)-domain-15(TBC1D15), is the GTPase-activating protein (GAP) for Ras-related Protein-7(RAB7) [13, 14] and an important molecular for lysosomal membrane regeneration [15-17]. During LLOMe-induced lysosomal damage, lysosomes swell, and their volume increases, while the levels of TBC1D15 are significantly elevated. Since TBC1D15 is involved in the repair and regeneration of lysosomal membrane damage, this suggests that the repair process is incomplete. When TBC1D15 is knocked down or its expression is inhibited, LLOMe-induced lysosomal volume swelling is significantly alleviated [18]. Since knocking down TBC1D15 reduces its role in lysosomal membrane repair, leading to a decrease in lysosomal volume [15], this suggests that TBC1D15 may be involved in the formation of increased lysosomal volume during swelling, and that lysosomal swelling is TBC1D15-dependent. In AD, lysosomal swelling is observed in microglial cells [19], suggesting that TBC1D15 may play a key role in Aβ-induced lysosomal swelling. In the LPS-induced intestinal inflammation model, upregulation of TBC1D15 can downregulate the expression of RAB7, cathepsin B, and Caspase3, thereby improving lysosomal function [20]. In an acute myocardial ischemia model, overexpression of TBC1D15 enhances autophagy, mitophagy, and lysosomal swelling [21]. However, overexpression of TBC1D15 can induce late-stage autophagy through miRNA-1, inhibiting RAB7 activity [22]. In AD animal models, inhibiting the expression of TBC1D15 enhances mitophagy and improves the morphology of neurons [18]. These findings suggest that, in the acute model, TBC1D15 levels are elevated, enhancing autophagy, while in the chronic model, TBC1D15 levels are reduced, impairing autophagy. Previous studies have shown that autophagy is enhanced in microglia during the early stages of AD [23, 24], while autophagy is impaired in microglia during the late stages of AD [23, 25]. By analogy, this strongly suggests that TBC1D15 may play an important role in the impaired autophagy of microglia in AD. Therefore, TBC1D15 may play a key role in lysosomal swelling and impaired autophagy in microglia in AD, but no relevant reports have been found to date.

In the present study, we will explore the dynamic changes in autophagy across different stages and cell types in AD. Additionally, we will observe the pH values and the degree of lysosomal swelling in Aβ-treated microglia under varying temporal and concentration gradients. The levels of TBC1D15 in microglia will be assessed at different stages of AD. Importantly, we will investigate whether the intervention of TBC1D15 can improve lysosomal swelling and enhance autophagy. Our study aims to identify a potential target for promoting Aβ clearance through the enhancement of autophagy.

## MATERIALS AND METHODS

### Animals

Male 3xTg AD mice were obtained from JAX Labs and underwent genotyping. All animals were housed at 24 ± 1.5°C in a specific pathogen-free (SPF) environment with unrestricted access to food and water. All animal experiments were conducted with random grouping and were approved by the Ethics Committee of Renmin Hospital, Wuhan University (No.20241101D).

### Aβ oligomers preparation and treatment of cells

1 mg Aβ peptide (A-42-T-5, GenicBio) was dissolved in 1 ml PBS to 205 μM. Next, the Aβ solution is placed in a shaking incubator at 37°C with a speed of 220 rpm and incubated for 24 hours. After incubation, the oligomerized Aβ is ultrasonicated on ice for 10 minutes to create a uniform Aβ oligomer solution and dilute the solution to 2, 5, 10, and 15 µM with culture medium during the treatment of cells [26-28].

### Western blot

After isoflurane anesthesia, the mice were sacrificed, and the brains were got out of the skull. The hippocampus was separated and homogenized in an ice-cold RIPA buffer containing protease inhibitors. Centrifuging at 12,000 rpm for 15 minutes at 4°C, the supernatant was collected, and protein concentration was measured using BCA assay. The proteins were resolved in a 10% SDS-polyacrylamide gel. The proteins were transferred from the gel to the membrane. Then the membrane was blocked for 1 hour at room temperature. Next, the membrane was incubated with primary antibody overnight at 4°C. After washing with TBST, the membrane was incubated with a secondary antibody conjugated to HRP for 1 hour at room temperature. After washing with TBST, the membrane was developed using an enhanced chemiluminescence (ECL), and BioRad digital imaging system was used to visualize protein bands, and the band intensity was analyzed [29-32]. All primary and secondary antibodies used in western blot were listed as following: P62/SQSTM1 Antibody (T55546S, Abmart Shanghai Co., Ltd.), LC3B Antibody (T55992S, Abmart Shanghai Co., Ltd.), Beta Actin Monoclonal antibody (66009-1-Ig, Proteintech Group, Inc), TREM2 Rabbit Polyclonal Antibody (AF8229, Beyotime), TBC1D15 Polyclonal antibody (17252-1-AP, Proteintech Group, Inc), Galectin3 Recombinant Rabbit Monoclonal Antibody (ET1702-48, HUABIO), LIMP II Recombinant Rabbit Monoclonal Antibody (HA721967, HUABIO), RAB7 Recombinant Rabbit Monoclonal Antibody (ET1611-96, HUABIO), Dynamin 2 Recombinant Rabbit Monoclonal Antibody(ET1705-2, HUABIO), RNF13 antibody (A8363, ABclonal), GAPDH Monoclonal antibody (60004-1-Ig, Proteintech Group, Inc), HRP conjugated Goat Anti-Mouse IgG (H+L) (GB23301, Servicebio), HRP conjugated Goat Anti-Rabbit IgG (H+L) ( GB23303, Servicebio).

### Immunofluorescence

The mice were anesthetized by isoflurane, and the brain was perfused with a fixative of 4% formaldehyde. After overnight post-fixation, the brain was embedded in paraffin to be sliced for 4 μm. The slice was blocked with donkey serum (BL939A, Biosharp) for 1 h at room temperature. Then, the slices were incubated with primary antibody at 4° for 24 h. The slice was washed 3 times with PBS containing 1% triton X-100 to remove any unbound primary antibody. Next, the slice was incubated with secondary antibody binding the fluorescence dye for 1 h at room temperature. For the three primary antibody incubation, every primary antibody was incubated with secondary antibody. The DAPI (BMD0063, Abbkine Scientific Co., Ltd) label nucleus for 15 min and the slide was covered with a mounting medium containing a fluorescent anti-fade reagent. Microscopy: The slice was observed under a fluorescence confocal microscope at specific wavelengths when excited to visualize the specific proteins, and the fluorescence intensity was measured for analysis [30]. A fluorescent secondary antibody is used as a negative control to detect if the antibody is specific. All primary and secondary antibodies used in immunofluorescence were listed as following: P62/SQSTM1 Antibody (T55546S, Abmart Shanghai Co., Ltd.), LC3B Antibody (T55992S, Abmart Shanghai Co., Ltd.), TBC1D15 Polyclonal antibody (17252-1-AP, Proteintech Group, Inc), Galectin3 Recombinant Rabbit Monoclonal Antibody (ET1702-48, HUABIO), LIMP II Recombinant Rabbit Monoclonal Antibody (HA721967, HUABIO), Ubiquitin Antibody (T55965S, Abmart Shanghai Co.,Ltd.), LAMP1 Recombinant Rabbit Monoclonal Antibody (HA722302,HUABIO), Purified anti-β-Amyloid, 1-16 Antibody(803004, BioLegend), iFluor™ 488 Conjugated Iba1 Recombinant Rabbit Monoclonal Antibody (HA720158F, HUABIO), Anti-GFAP Rabbit pAb (GB11096, Servicebio), CoraLite® Plus 488-conjugated NeuN Polyclonal antibody (CL488-26975. Proteintech Group, Inc), RNF13 antibody (PC9158, Abmart Shanghai Co., Ltd), Alexa Fluor® 594 AffiniPure™ F(ab')_2_ Fragment Donkey Anti-Rabbit IgG (H+L)(711-586-152, Jackson ImmunoResearch Inc.), Alexa Fluor® 647 AffiniPure™ F(ab')_2_ Fragment Donkey Anti-Rabbit IgG (H+L)(711-606-152, Jackson ImmunoResearch Inc.), Alexa Fluor® 488 AffiniPure™ F(ab')_2_ Fragment Donkey Anti-Rabbit IgG (H+L)( 711-546-152, Jackson ImmunoResearch Inc.), Alexa Fluor® 594 AffiniPure™ F(ab')_2_ Fragment Donkey Anti-Mouse IgG (H+L)( 715-586-150, Jackson ImmunoResearch Inc.), Actin-Tracker Red-594(C2205S, Beyotime).

### Cell culture

BV2 cell is a microglial cell line derived from mice. HT-22 cell is a neuronal cell line from mouse hippocampal. C8-D1A cell is an astrocyte cell line derived from mice. BV2 cells, HT-22 cells, and C8-D1A cells were obtained from Wuhan Pricella Biotechnology (Wuhan, China) cultured in DMEM, containing 10% fetal bovine serum, 1% penicillin-streptomycin solution, at 37°C in a humidified incubator with 5% CO_2_.

### Cell transfection

For plasmids transfection, the pEnCMV-mCherry-mLC3-EGFP-SV40-Neo and pCMV-mLAMP1-AT 1.03 plasmids were obtained from Wuhan Qinda Biological technology Co., LTD. Bacteria carrying the plasmids were placed in Luria-Bertani medium and cultured in a constant temperature shaker at 37°C overnight. The plasmids were extracted using the TIANprep Mini Plasmid Kit (DP103, TIANGEN) and subsequently used to transfect cells. Cells were inoculated at a suitable density (60-70%) in a 35 mm glass-bottom dish the day before transfection (18-24 h) and transfected using Lipo6000™ Transfection Reagent (C0526, Beyotime). 5 μl of transfection reagent was added to each dish, and 2.5 ug of empty plasmid and pEnCMV-mCherry-mLC3-EGFP-SV40-Neo and pCMV-mLAMP1-AT 1.03 plasmids were added. After 6 hours of transfection, the culture medium was replaced with a fresh complete medium, and then the cells were placed in a 5% CO2 incubator for 18 hours. Then Aβ was added for different stimulation interventions. Finally, the cells were observed under a laser confocal microscope [33].

For siRNA transfection, siRNAs were synthesized from Wuhan Qinda Biological technology Co., LTD. Transfections were performed in six-well and 12-well plates, inoculated at 50% density into well plates for incubation the day before transfection (18-24 hours). The siRNA concentration was diluted to 20 µM, and for six-well plates, 5 µl of Lipo6000™ Transfection Reagent (C0526, Beyotime) was added to each well, and 100 pM of both siRNA control and siRNA TBC1D15, siRNA LIMP II, and siRNA ATG8 were added to each well, and for the 12-well plates, the above amounts were halved. DMEM was used for transfection, and after 6 hours of transfection, the medium was replaced with fresh complete medium, and incubation was continued for 24 hours, Then Aβ was added to different groups, at last, cellular proteins were extracted for western blot and fixed for cellular immunofluorescence at the end of the treatment time.

### Stereotaxic injection

After anesthetizing the mice with isoflurane, the head is positioned in the stereotactic frame, ensuring the skull surface is level. The skin on the head is incised to expose the skull. The bregma is located and marked. Using the bregma as a reference point, the injection coordinates for the prefrontal cortex are AP: +2.0 mm, ML: ±0.5 mm, DV: -1.5 mm. Two microliters of the AAV-IBA1-ShRNA-TBC1D15 virus are injected into the prefrontal cortex. After the injection is completed slowly, the needle is kept in place for 5 minutes before being withdrawn slowly. The wound is disinfected, and the skin is sutured. After the mice recover, they are continued to be raised for 2 weeks, followed by slice observations of amyloid plaques [34].

### Analysis of Aβ phagocytosis capability

1.0% NH_4_OH was added directly to FAM-labelled Aβ (AS-23526-01, Anaspec) (add 35-40 μl to 0.5 mg or 70-80 μl to 1 mg). The peptide cannot be stored in 1.0% NH_4_OH for long periods, so immediately dilute the solution with PBS to a concentration of approximately 1 mg/ml, then dispense into individual tubes for storage at -80°C. For use, the solution was diluted with complete medium to a working concentration of 0.5 μM, aggregated in a shaking incubator at 37°C with a speed of 220 rpm incubated for 1 hour, and then ultrasonicated for 10 min, followed by treatment of the cells. Then, the oligomerized Aβ solution was added to BV2 cells growing on the glass slide for different times of incubation and phagocytosis. After finishing incubation, the cells were washed with PBS to remove the residual oligomers. Then, the phalloidin solution (C2205S, Beyotime) was added to the cells for 30 minutes to stain the cytoskeleton, and after the slides were re-washed with PBS, the DAPI solution was added to the slide to label the nucleus. Finally, the slides were mounted with an antifade mounting medium for observation under confocal microscopy. The Aβ puncta were counted to evaluate the phagocytosis capability [35].

### pH analysis of autophagic lysosome

Red fluorescent protein (mCherry) maintains a stable signal in acidic environments. In the acidic environment of autophagic lysosomes, GFP fluorescence quickly quenches. After transfection with the pEnCMV-mCherry-mLC3-EGFP-SV40-Neo plasmid, GFP fluorescence rapidly quenches, leaving only red fluorescent signals, indicating that autophagic lysosomes exhibit red fluorescence. When autophagic lysosomes are inhibited, yellow fluorescence increases, reflecting an elevated pH within the lysosomes and reduced degradation of lysosomal contents. This is used to assess changes in autophagic lysosomal pH [36].

### Analysis of lysosome volume

The cells were stained with lysotracker (C1046, Beyotime) and Hoechst 3342 (C1025, Beyotime) for 30 min. After washing the cells with PBS, cells were observed under a laser confocal microscope. The volume of the lysotracker labeled puncta was measured to evaluate the extent of lysosomal swelling.

**Figure 1.**
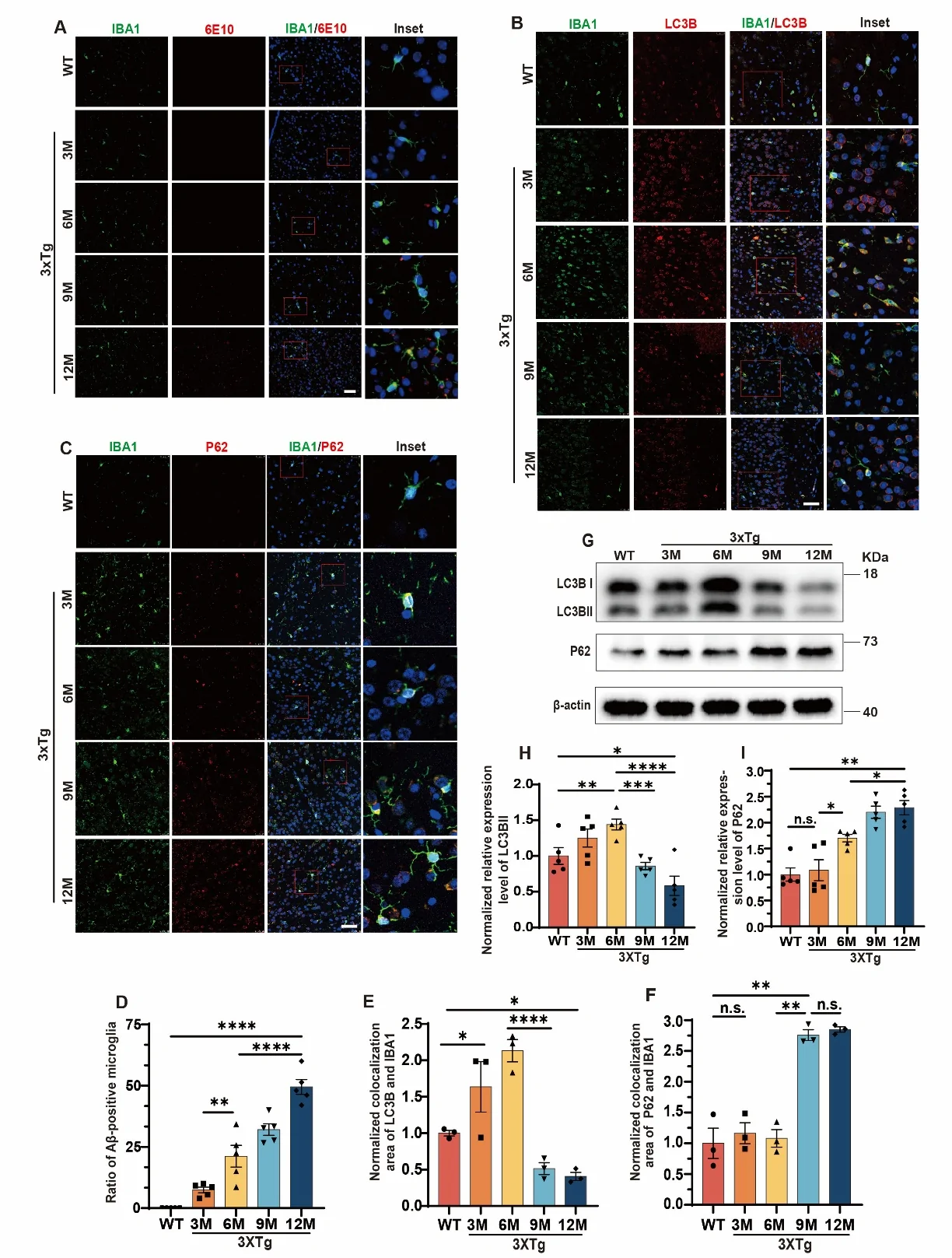
**Dynamics of microglial autophagy in 3xTg AD mice at different ages**. (**A**) The levels of Aβ phagocytosis by microglia in wild-type, 3-month-old, 6-month-old, 9-month-old, and 12-month-old 3xTg AD mice. n=5/group, scale bars: 50 μm. (B, C) Immunofluorescence was used to observe the distribution levels of LC3B and P62 in microglial cells of 3xTg AD mice at different ages. n=3/group, scale bars: 50 μm. (**D**) Aβ-positive area in microglia at different ages. n=5/group. (E, F) Normalized analysis of the LC3B-positive particle area and the P62-positive particle area in microglia of AD mice at different ages. n=3/group. (**G**) Western blot analysis of LC3B levels in wild-type and AD mice of different ages. n=5/group. (H, I) Analysis of the relative expression levels of LC3B and P62 in Western blot. n=5/group. Data from all experiments are expressed as mean ± standard error of the mean (SEM). Nonparametric tests for two independent samples were performed with the Wilcoxon rank-sum test. For nonparametric tests between multiple groups, the Kruskal-Wallis test was used. Significance levels are indicated by p-values < 0.05(*), p < 0.01(**), p < 0.001(***) and p < 0.0001(****).

**Figure 2.**
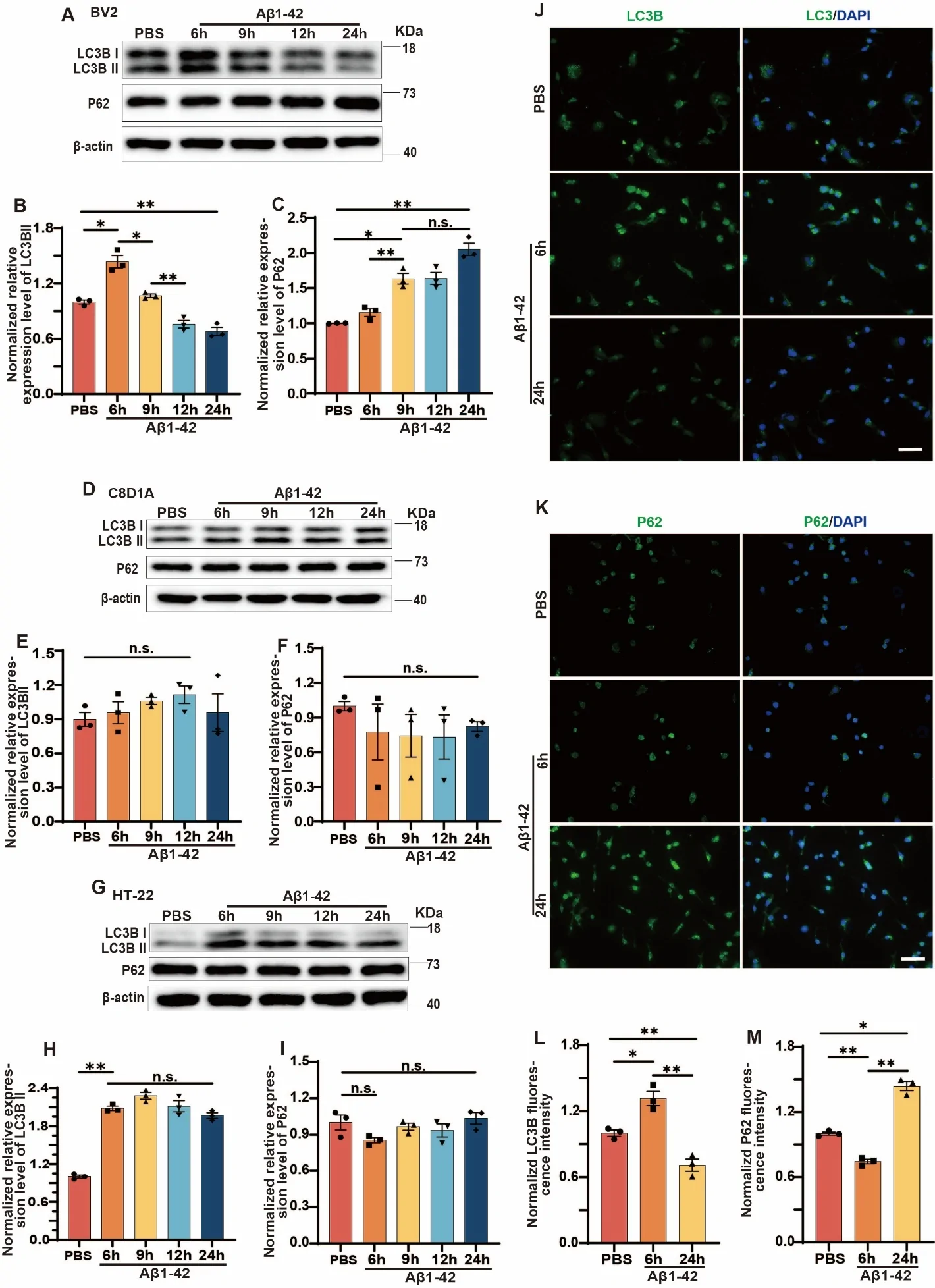
**BV2 cells exhibit time-dependent dynamic changes in autophagy following Aβ treatment**. (**A**) BV2 cells were treated with Aβ oligomers for 6, 9, 12, and 24 h, and controls were treated with PBS, and Western blot was used to detect the levels of LC3B and P62, with β-actin as a loading control. n=3/group. (B, C) Relative quantification of LC3B and P62 levels was performed. n=3/group. (D, E, F) C8D1A cells received corresponding treatments, and the levels of LC3B and P62 were detected with relative quantification analysis. n=3/group. (G, H, I) HT22 cells were subjected to similar treatments to detect LC3B and P62 levels, followed by relative quantification analysis. n=3/group. (J, K, L, M) BV2 cells were treated with Aβ oligomers for 6 and 24 h, and controls were treated with PBS. Immunofluorescence was utilized to observe the levels of LC3B and P62 in BV2 cells, and the relative intensity of LC3B and P62 immunofluorescence was analyzed. n=3/group, scale bars: 50 μm. Data from all experiments are expressed as mean ± standard error of the mean (SEM). Nonparametric tests for two independent samples were performed with the Wilcoxon rank-sum test. For nonparametric tests between multiple groups, the Kruskal-Wallis test was used. Significance levels are indicated by p-values < 0.05(*), p < 0.01(**).

### Energy metabolism analysis of lysosome

The pCMV-mLAMP1-AT 1.03 protein is an ATP fluorescence probe based on fluorescence resonance energy transfer (FRET), designed for real-time monitoring of ATP levels and dynamics in lysosome of single living cells. It consists of N-terminal cyan fluorescent protein (mseCFPΔC11)(Excitation at 435 nm, emission at 475 nm), ε subunit of Bacillus subtilis FoF1-ATP synthase (Asmall ATP-binding protein (~14 kDa) with an N-terminal β-barrel domain and two C-terminal α-helix domains), the ε subunit specifically binds ATP without hydrolyzing it or binding ADP, GTP, CTP, or UTP. The C-terminal is a yellow fluorescent protein (cp173-mVenus) (Excitation at 515 nm, emission at 527 nm). In pCMV-mLAMP1-AT 1.03, ATP binding to the ε subunit induces significant conformational changes, bringing the cyan and yellow fluorescent proteins close together. This proximity produces a FRET effect, transferring excitation energy from the cyan to the yellow fluorescent protein, enabling real-time detection of millimolar ATP levels in lysosome [18, 37].

### Statistical Analysis

Statistical analyses were performed using GraphPad Prism software (GraphPad Software, La Jolla, CA). Data from all experiments are expressed as mean ± standard error of the mean (SEM). Normal testing was performed using the Shapiro-Wilk test. For variables that followed a normal distribution, multiple-group mean comparisons were analyzed by one-way ANOVA with the Turkey post hoc test. Non-parametric tests were used for data that did not follow a normal distribution. Nonparametric tests for two independent samples were performed with the Wilcoxon rank-sum test. For nonparametric tests between multiple groups, the Kruskal-Wallis test followed by Dunn’s multiple comparison test was used. Significance levels are indicated by p-values p< 0.05 (*), p < 0.01 (**), p < 0.001 (***) and p < 0.0001 (****) [38, 39].

## RESULTS

### Microglial autophagy in AD showed the dynamical trend from enhancement to decrease

In 3XTg AD mice, the engulfment of Aβ by microglia increased from 3 months to 12 months of age (p < 0.01) (Fig. 1A, 1D), indicating a sustained enhancement in the engulfment capacity of microglia. The Aβ engulfed by microglia needs to be degraded as waste in order to facilitate the engulfment of additional Aβ or other metabolic byproducts through autophagy. However, whether microglia exhibit a similar trend in autophagy remains unclear. To elucidate the exact trend of autophagy in microglia, we assessed autophagy in wild-type mice and 3xTg AD mice at 3, 6, 9, and 12 months of age, utilizing immunofluorescence staining of light chain 3B (LC3B) and Sequestosome 1 (SQSTM1/P62), two critical markers for autophagy (Fig. 1B-1C). The data revealed that LC3B levels increased from 3 to 6 months of age (p < 0.05) but decreased from 9 to 12 months in 3xTg AD mice compared to wild-type mice (p < 0.05) (Fig. 1E). Concurrently, P62 levels increased from 9 to 12 months in 3xTg AD mice compared to wild-type mice (p < 0.01) (Fig. 1F). To further validate the trend of autophagy, we analyzed the levels of LC3B and P62 using Western blot. The results demonstrated that LC3BII levels increased from 3 to 6 months in 3xTg AD mice compared to wild-type mice (p < 0.01) (Fig. 1G, 1H). Additionally, P62 levels increased from 9 to 12 months in 3xTg AD mice compared to wild-type mice (p < 0.01) (Fig. 1G, 1I). These findings suggest that autophagy increased from 3 to 6 months and gradually continued to rise from 9 months onward.

To investigate whether only microglia exhibited this autophagy trend, BV2, C8D1A, and HT22 cells were treated with oligomerized Aβ for 6, 9, 12, and 24 hours, and autophagy was assessed (Fig. 2A, 2D, 2G). The analysis indicated that LC3B levels increased at 6 hours post-Aβ treatment in BV2 cells (p < 0.05), returned to baseline levels at 9 hours, and decreased at 12 hours (p < 0.01) (Fig. 2A, 2B). P62 levels began to increase at 9 hours post-Aβ treatment (p < 0.05) (Fig. 2C), and this was confirmed through immunofluorescence staining analysis (Fig. 2J, 2K, 2L, 2M). In contrast, LC3B and P62 levels in C8D1A cells did not show significant increases following Aβ treatment (p > 0.05) (Fig. 2E, 2F). Interestingly, while LC3B levels rose significantly from 6 to 24 hours post-Aβ treatment (p < 0.01) (Fig. 2H), P62 levels did not exhibit a significant increase (p > 0.05) (Fig. 2I). These results suggest that only microglial autophagy in vitro mirrored the trends observed in AD mice following Aβ treatment.

**Figure 3.**
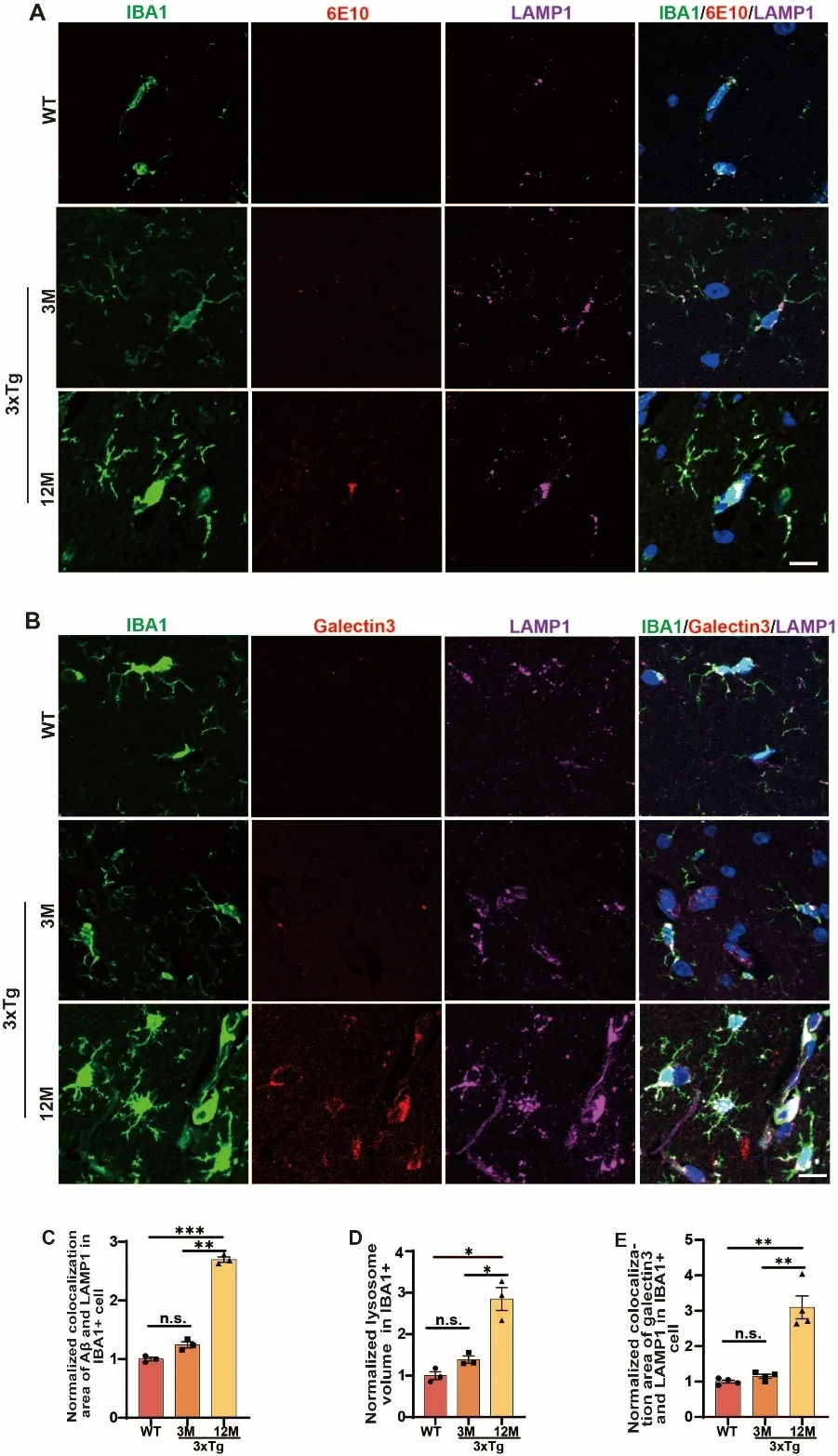
**With increasing age, 3xTg mice exhibit enhanced Aβ phagocytosis by microglial cells, along with enlarged lysosomal volume and aggravated damage**. (**A**) Brain slices from wild-type, 3-month-old, and 12-month-old mice were incubated with IBA1 antibody (green), 6E10 antibody (red), and LAMP1 antibody (purple) to observe microglial phagocytosis of plaques and lysosomal morphology. n=3/group. Scale bar=20 μm. (**B**) The slices were also incubated with IBA1 antibody (green), galectin3 antibody (red), and LAMP1 antibody (purple) to assess lysosomal damage in microglia. n=4/group. Scale bar=25 μm. (**C**) In IBA1-labeled cells, positive particles co-localizing with 6E10 and LAMP1 were statistically analyzed. n=3/group. (**D**) The area of LAMP1-positive particles in IBA1-labeled cells was quantified. n=3/group. (**E**) In IBA1-labeled cells, positive particles co-localizing with galectin3 and LAMP1 were also statistically analyzed. n=4/group. Data from all experiments are expressed as mean ± standard error of the mean (SEM). Nonparametric tests for two independent samples were performed with the Wilcoxon rank-sum test. For nonparametric tests between multiple groups, the Kruskal-Wallis test was used. Significance levels are indicated by p-values < 0.05(*), p < 0.01(**), p < 0.001(***).

### The decreasing microglia autophagy concomitant with impaired lysosome in AD mice

Since lysosomes are the executors of autophagy, we aimed to investigate the reasons behind the observed decrease in autophagy in microglia by analyzing lysosomal structure through immunofluorescence. Upon Aβ phagocytosis, the volume of microglial lysosomes increased, particularly at 12 months compared to 3 months in AD mice (p < 0.05) (Fig. 3A, 3C, 3D). Galectin3, a marker for damaged lysosomes, revealed that lysosomal damage in microglial cells from 12-month AD mice was significantly greater than that in 3-month AD mice (p < 0.01) (Fig. 3E). Given the decreased autophagy observed in 12-month AD mice, these results suggest that the reduction in autophagy is associated with impaired lysosomal function in the microglia of AD mice.

**Figure 4.**
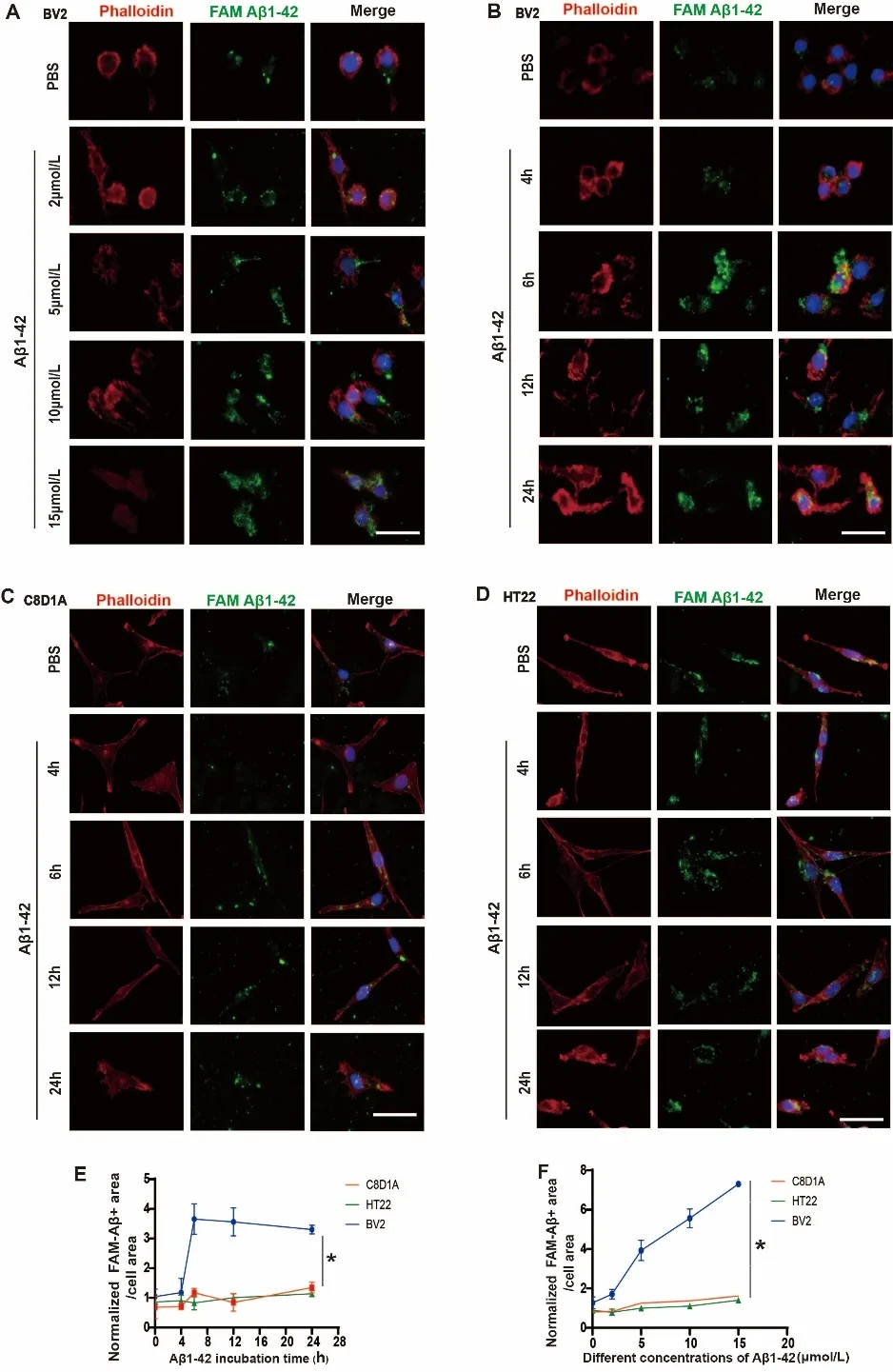
**BV2 cells, rather than C8D1A and HT22 cells, exhibit an increase in phagocytosis that is dependent on time and Aβ concentration**. (**A**) BV2 cells were treated with Aβ oligomers at concentrations of 2 μM, 5 μM,10 μM, and 15 μM, and the control group was treated with PBS, and then BV2 phagocytosis was measured with FAM-labeled Aβ oligomers. n=3/group, scale bar=50 μm. (**B**) BV2 cells were treated with 10 μM Aβ oligomers and incubated for 4 h, 6 h, 12 h, and 24 h. Controls were treated with PBS, and their phagocytosis of Aβ oligomers was observed with FAM-labeled Aβ oligomers. n=3/group, scale bar=50 μm. (C, D) C8D1A cells and HT22 cells were treated with 10 μM Aβ oligomers and incubated for 4 h, 6 h, 12 h, and 24 h. Controls were treated with PBS, and their phagocytosis of Aβ oligomers was observed with FAMlabeled Aβ oligomers. n=3/group, scale bar=10 μm. (**E**) A relative quantitative analysis of Aβ phagocytosis levels at different concentrations was performed for the three cell types. n=3/group. (**F**) A relative quantitative analysis of Aβ phagocytosis levels at the different incubation times was conducted for the three cell types. n=3/group. Data from all experiments are expressed as mean ± standard error of the mean (SEM). Non-parametric tests were used for data that did not follow a normal distribution. Nonparametric tests for two independent samples were performed with the Wilcoxon rank-sum test. For nonparametric tests between multiple groups, the Kruskal-Wallis test followed by Dunn’s multiple comparison test was used. Significance levels are indicated by p-values < 0.05(*).

**Figure 5.**
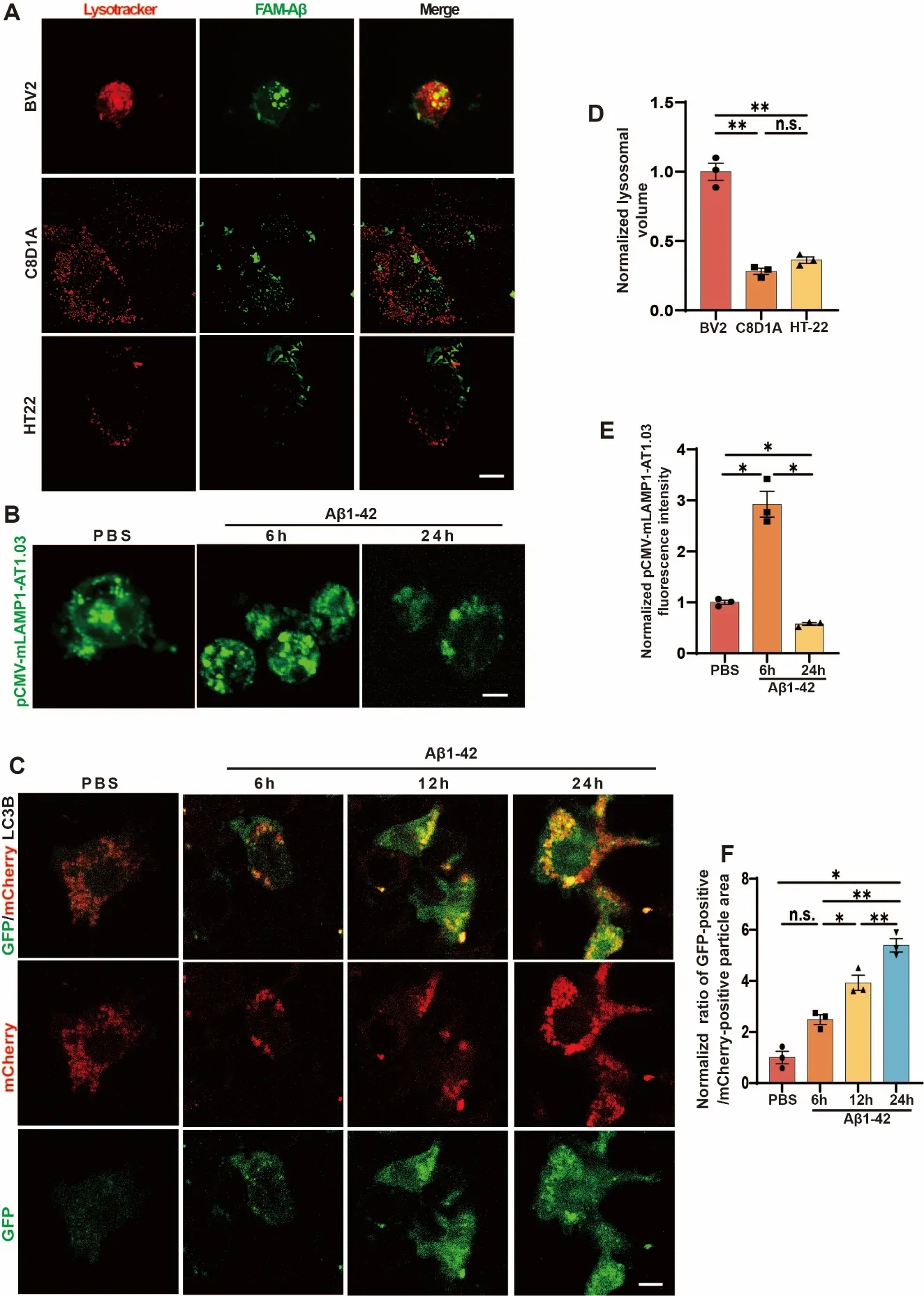
**BV2 cells, rather than C8D1A cells and HT22 cells, show lysosomal swelling and damage after Aβ phagocytosis**. (**A**) BV2 cells, C8D1A, and HT22 cells were incubated with 10 μM Aβ, and lysotracker staining was used to observe lysosomal volume. n=3/group, scale bar=10 μm. (**B**) BV2 cells were treated with 10 μM Aβ oligomers and incubated for 6 and 24 h. Controls were treated with PBS. An ATP-sensitive AT 1.03-LAMP1 protein was used to detect ATP levels within lysosomes. n=3/group, scale bar=10 μm. (**C**) BV2 cells were treated with 10 μmol/L Aβ oligomers and incubated for 6 h, 12 h, and 24 h. Controls were treated with PBS. The GFP-mCherry-LC3 protein was employed to assess the pH levels inside lysosomes. n=3/group, scale bar=10 μm. (**D**) After Aβ oligomer treatment of BV2 cells for 6 hours and 24 hours, the area of lysotracker-positive particles was normalized for analysis. n=3/group. (**E**) After Aβ oligomer treatment of BV2 cells for 6 h and 24 h, the fluorescence intensity of AT1.03-LAMP1 protein was normalized for analysis. n=3/group. (**F**) Aβ oligomer treatment of BV2 cells for 6, 12, and 24 h were analyzed by normalizing the ratio of GFP-positive particle area to mCherry-positive particle area. n=3/group. Data from all experiments are expressed as mean ± standard error of the mean (SEM). Non-parametric tests were used for data that did not follow a normal distribution. Nonparametric tests for two independent samples were performed with the Wilcoxon rank-sum test. For nonparametric tests between multiple groups, the Kruskal-Wallis test followed by Dunn’s multiple comparison test was used. Significance levels are indicated by p-values < 0.05(*), p < 0.01(**).

To determine whether lysosomal damage in microglia correlates with Aβ phagocytosis, we treated BV2, C8D1A, and HT22 cells with varying concentrations and durations of Aβ (Fig. 4A, 4B, 4C, 4D). The data showed that Aβ phagocytosis increased in microglia with higher concentrations and longer treatment times (Fig. 4E, 4F). Additionally, the lysosomal volume increased exclusively in microglia, with no significant changes observed in astrocytes or neurons (Fig. 5A, 5D). These findings suggest a close relationship between decreased autophagy and increased lysosomal volume. Given the importance of energy and optimal pH for lysosomal function, we further investigated whether the swelling of lysosomes induced by Aβ affected their functionality. ATP levels and pH values in microglial lysosomes were analyzed using pCMV-mLAMP1-AT 1.03 and GFP-mCherry-LC3B plasmid transfection (Fig. 5B, 5C). The analysis revealed that Aβ treatment significantly reduced lysosomal ATP levels and increased pH values in microglia (p < 0.05) (Fig. 5E, 5F). Meanwhile, there was no significant change in lysosomal pH values and lysosomal ATP levels in HT-22 and C8D1A cells (Supplementary Fig. 1A-D).

### Aβ phagocytosis of microglia had interacted with autophagy

**Figure 6.**
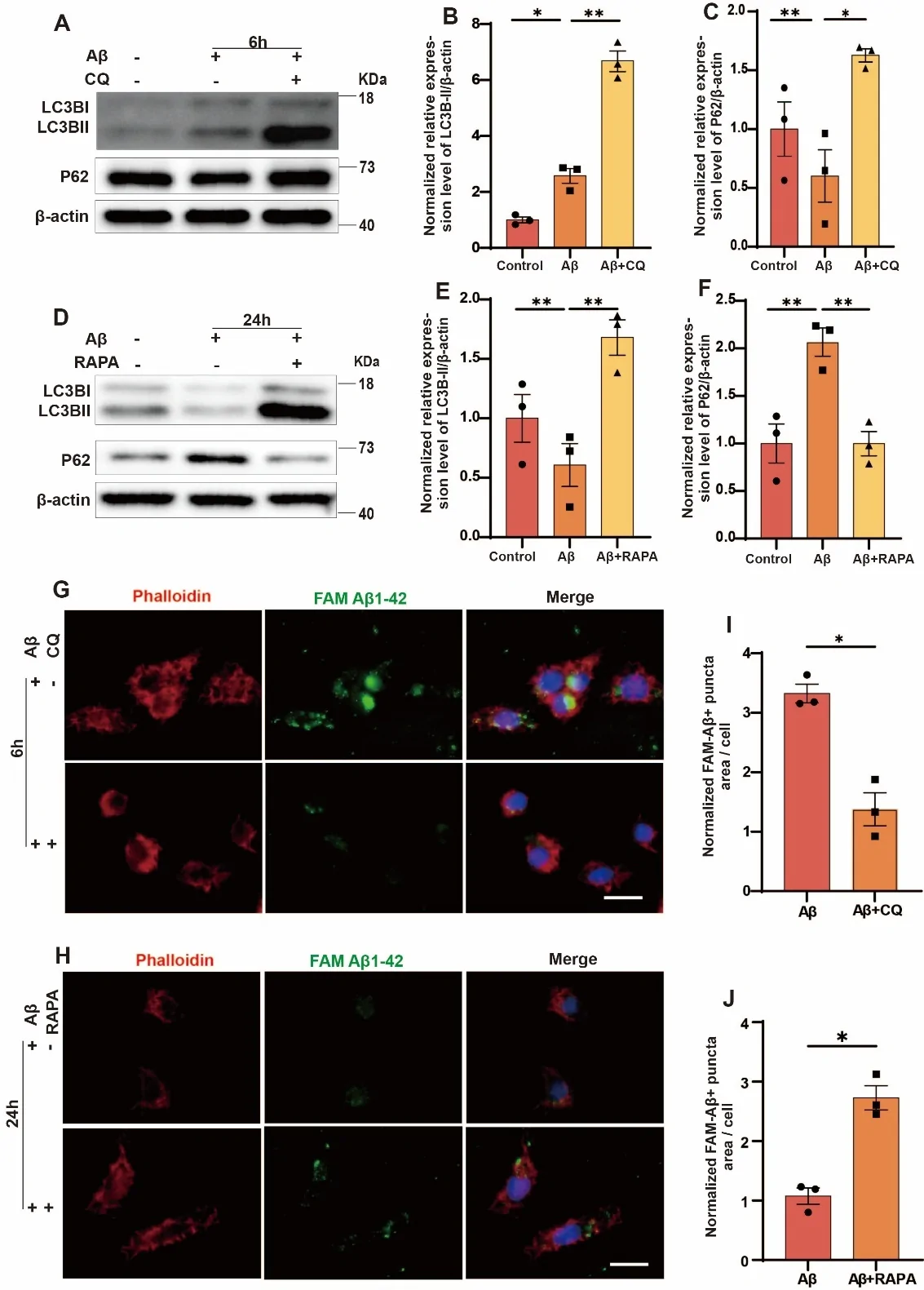
**Aβ treatment significantly impairs the autophagy flux and phagocytic ability of BV2 cells**. (**A**) BV2 cells were treated with Aβ or Aβ combined with chloroquine for 6h, and controls were treated with PBS. And the levels of LC3B and P62 were detected using western blot analysis. n=3/group. (B, C) A relative quantitative analysis of the levels of LC3B and P62 was conducted. n=3/group. (**D**) BV2 cells were treated with Aβ or Aβ combined with rapamycin for 24h. Controls were treated with PBS. And the levels of LC3B and P62 were detected using western blot analysis. n=3/group. (E, F) A relative quantitative analysis of the levels of LC3B and P62 was performed. n=3/group. (G, H) BV2 cells were treated with FAM-labeled Aβ or with chloroquine and rapamycin, to observe phagocytosis of Aβ. n=3/group, scale bar=25 μm. (I, J) A relative quantitative analysis of Aβ levels within BV2 cells was conducted. n=3/group. Data from all experiments are expressed as mean ± standard error of the mean (SEM). Non-parametric tests were used for data that did not follow a normal distribution. Nonparametric tests for two independent samples were performed with the Wilcoxon rank-sum test. For nonparametric tests between multiple groups, the Kruskal-Wallis test followed by Dunn’s multiple comparison test was used. Significance levels are indicated by p-values < 0.05(*), p < 0.01(**).

To further investigate the effect of Aβ-induced lysosomal damage on autophagy flux in microglia, BV2 cells were treated with oligomerized Aβ, and levels of LC3B and P62 were assessed using Western blot analysis. The results demonstrated that after 6 hours of Aβ treatment, LC3B levels increased while P62 levels decreased, and this effect was inhibited by chloroquine (p < 0.05) (Fig. 6A, 6B, 6C). Conversely, after 24 hours of Aβ treatment, LC3B levels decreased, and P62 levels increased, with rapamycin effectively inhibiting this change (p < 0.05) (Fig. 6D, 6E, 6F). These findings indicate that Aβ treatment initially inhibits autophagy flux at the 6-hour mark, followed by activation of autophagy flux at the 24-hour mark. This suggests a concurrent occurrence of lysosomal damage and autophagy inhibition, as the 6-hour Aβ treatment does not induce lysosomal damage, while the 24-hour treatment severely compromises lysosomal integrity. Further investigation revealed that the activation of autophagy flux after 24 hours of Aβ treatment enhances the phagocytosis of Aβ by microglial cells, whereas the inhibition of autophagy flux after 6 hours suppresses microglial phagocytosis of Aβ (Fig. 6G-6J). These results suggest a significant interaction between microglial autophagy and Aβ phagocytosis, highlighting that lysosomal damage is associated with the interplay between autophagy and phagocytosis.

### TBC1D15 level in microglial lysosome increased in 3xTg AD mice

**Figure 7.**
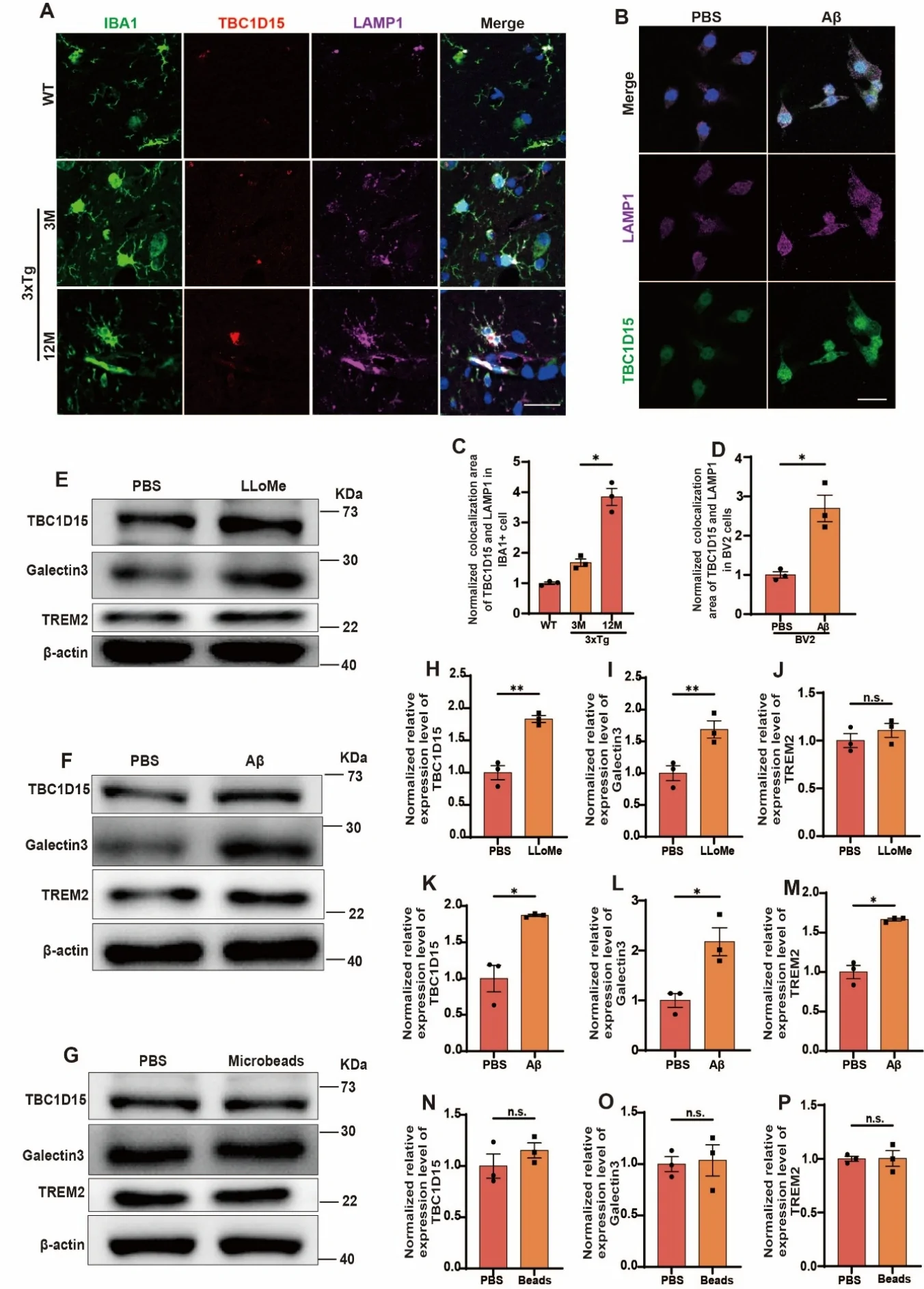
**In the microglial lysosomes of 3xTg AD mice, the expression level of TBC1D15 was significantly increased**. (**A**) Brain slices from wild-type, 3-month-old, and 12-month-old 3xTg AD mice were stained with antibodies against IBA1 (green), TBC1D15 (red), and LAMP1 (purple). n=3, scale bar=25 μm. (**B**) After treating BV2 cells with Aβ oligomers, controls were treated with PBS. The cells were stained for TBC1D15 (green) and LAMP1 (red). n=3/group, scale bar=25 μm. (**C**) The area of co-localized particles of TBC1D15 and LAMP1 in IBA1-positive cells was quantitatively analyzed. n=3/group (D) Similarly, the area of colocalized particles of TBC1D15 and LAMP1 in BV2 cells was also analyzed. n=3/group. BV2 cells were incubated separately with LLoMe (which can specifically damage lysosomal membranes), Aβ, and microbeads. (E, F, G) Cells were treated with LLoMe, Aβ and microbeads. The control group was treated with PBS. Western blot was employed to detect TBC1D15, galectin3 (a marker of lysosomal damage), TREM2 (a receptor mediating Aβ phagocytosis), and β-actin as a loading control. n=3/group. (H, I, J) TBC1D15, galectin3, and TREM2 were relatively quantified for LLoMe treatment. n=3/group. K, L, M. TBC1D15, galectin3, and TREM2 were relatively quantified for Aβ treatment. n=3/group. (N, O, P) TBC1D15, galectin3, and TREM2 were relatively quantified for microbeads treatment. n=3/group. Data from all experiments are expressed as mean ± standard error of the mean (SEM). Non-parametric tests were used for data that did not follow a normal distribution. Nonparametric tests for two independent samples were performed with the Wilcoxon rank-sum test. For nonparametric tests between multiple groups, the Kruskal-Wallis test followed by Dunn’s multiple comparison test was used. Significance levels are indicated by p-values < 0.05(*), p < 0.01(**).

TBC1D15 is a crucial protein involved in lysosomal membrane repair [15]. To investigate the key molecules associated with lysosomal damage in AD microglia, we assessed TBC1D15 levels using immunofluorescence techniques. In 3-month-old AD mice, TBC1D15 levels in microglial lysosomes were moderately elevated compared to wild-type mice. In contrast, 12-month-old AD mice exhibited a significant increase in TBC1D15 levels (Fig. 7A, 7C). Similarly, treatment with Aβ in BV2 cells resulted in a substantial increase in TBC1D15 levels (Fig. 7B, 7D), suggesting that elevated TBC1D15 may reflect lysosomal damage. LLOMe is a compound known to directly damage the lysosomal membrane, while microbeads serve as non-toxic controls. To clarify whether Aβ causes lysosomal damage in microglia, BV2 cells were treated with LLOMe, Aβ, and microbeads, it was found that the co-localization area of LAMP1 and galectin3 increased after LLOMe and Aβ treatment, indicating that these two treatments may damage lysosomes (Supplementary Fig. 2A-F). To further analyze the response of TBC1D15 to lysosomal damage, we compared the effects of Aβ, LLOMe, and microbeads on BV2 cells. The results indicated that both LLOMe and Aβ treatment led to significant increases in the levels of TBC1D15 and galectin3. Galectin3 is a recognized marker of lysosomal damage [40](Fig. 7E, 7F, 7H, 7I, 7K, 7L), whereas microbeads did not elicit such an effect (Fig. 7G, 7N, 7O). These suggest that elevated TBC1D15 may be involved in lysosomal damage. Additionally, since galectin3 is known to promote TREM2 expression [41], we observed that the increase in galectin3 levels induced by Aβ-rather than by LLOMe-resulted in a significant upregulation of TREM2 levels in microglial cells (Fig. 7J, 7M). Given that TREM2 mediates the phagocytic function of microglia, these results imply that even in the context of lysosomal damage induced by Aβ oligomers, the phagocytic activity of microglial cells remains elevated. This raises the possibility of a positive feedback loop between lysosomal damage and phagocytosis in microglial cells.

### Knockdown of TBC1D15 can improve the decline in autophagy and damaged lysosome induced by Aβ

**Figure 8.**
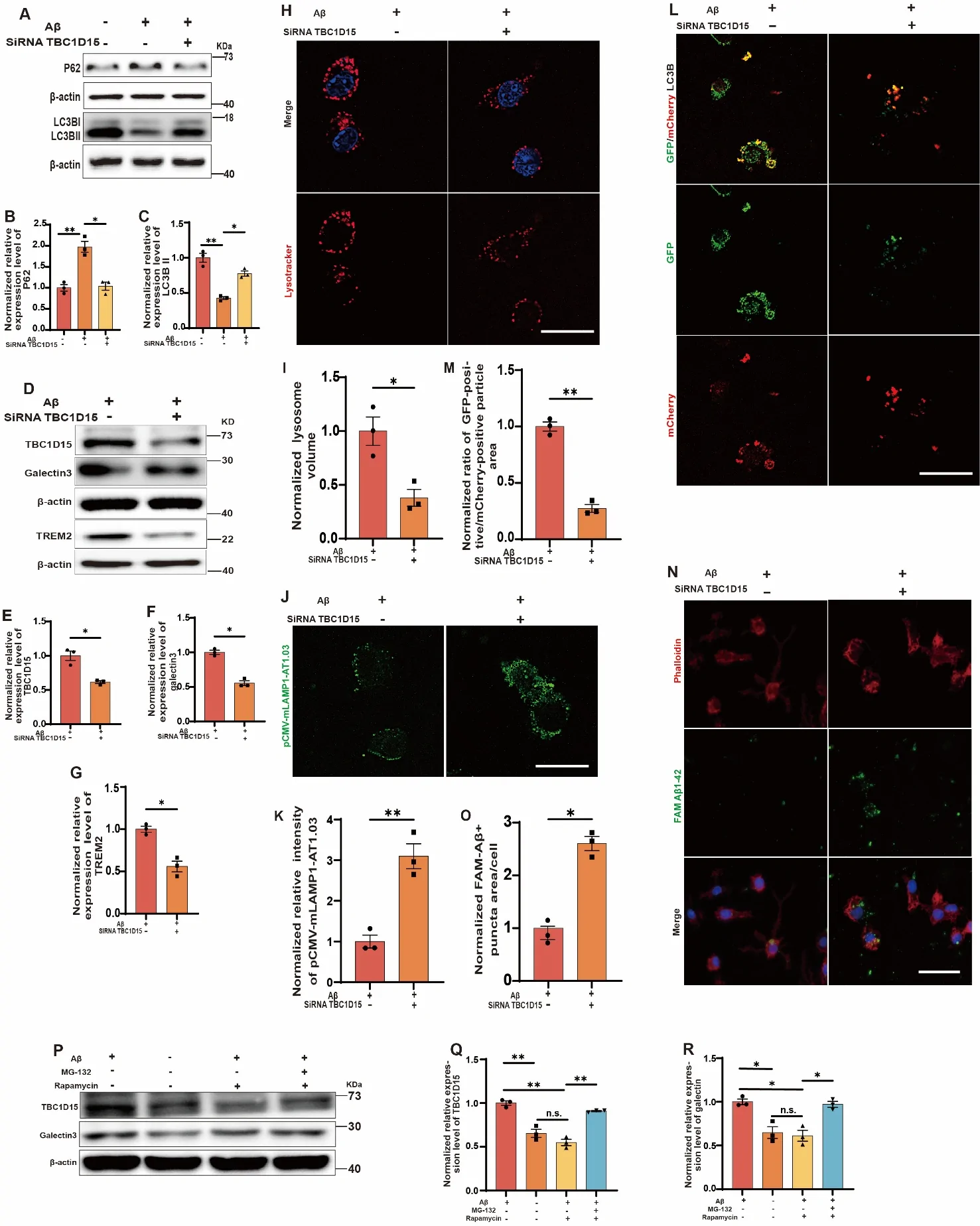
**Knockdown of TBC1D15 can improve the autophagy decline and lysosomal damage in BV2 cells treated with Aβ oligomers**. (**A**) BV2 cells were treated with Aβ oligomers or Aβ oligomers combined with siRNA TBC1D15. The control group was treated with PBS. LC3B and P62 protein levels were detected using Western blot analysis. n=3/group. (B, C) LC3, and P62 proteins were relatively quantified. D. BV2 cells were treated with Aβ oligomers or Aβ oligomers combined with siRNA TBC1D15. TBC1D15, TREM2, and Galectin3 protein levels were detected using Western blot analysis. n=3/group. (E, F, G) TBC1D15, TREM2, and Galectin3 proteins were relatively quantified. H. BV2 cells were treated with Aβ oligomers or Aβ oligomers combined with siRNA TBC1D15. Lysotracker was used to label lysosomes to observe lysosomal volume. N=3/group. Scale bars: 25 μm. I. Normalized analysis of lysosomal volume. n=3/group. (**J**) BV2 cells were treated with Aβ oligomers or Aβ oligomers combined with siRNA TBC1D15. The pCMV-mLAMP1-AT1.03 plasmid was transfected to assess lysosomal ATP levels. n=3/group. Scale bars: 25μm. (**K**) Normalized analysis of the area of pCMV-mLAMP1-AT 1.03-positive particles. n=3/group. (**L**) BV2 cells were treated with Aβ oligomers or Aβ oligomers combined with siRNA TBC1D15. The GFP-mCherry-LC3B plasmid was transfected to evaluate lysosomal pH levels. n=3/group. Scale bars: 25μm. (**M**) The ratio of fluorescence intensity GFP-positive particle to mCherry-positive particle was normalized for analysis. n=3/group. (**N**) BV2 cells were treated with Aβ oligomers or Aβ oligomers combined with siRNA TBC1D15. Phagocytosis experiments were conducted using FAM-labeled Aβ oligomers. n=3/group. Scale bars: 50 μm. (**O**) The area of Aβ oligomers in BV2 cells was normalized for analysis. n=3/group. (**P**) BV2 cells were treated with Aβ oligomers, rapamycin, or MG132. The control group was treated with PBS. Western blot was used to detect the protein levels of TBC1D15 and galectin3. n=3/group. (Q, R) Relative quantification analysis of TBC1D15 and galectin3 protein levels. n=3/group. Data from all experiments are expressed as mean ± standard error of the mean (SEM). Non-parametric tests were used for data that did not follow a normal distribution. Nonparametric tests for two independent samples were performed with the Wilcoxon rank-sum test. For nonparametric tests between multiple groups, the Kruskal-Wallis test followed by Dunn’s multiple comparison test was used. Significance levels are indicated by p-values < 0.05(*), p < 0.01(**).

Since lysosomal damage is correlated with autophagy decrease, and TBC1D15 serves as a marker for lysosomal damage, we aimed to investigate whether TBC1D15 is involved in regulating microglial autophagy changes following Aβ treatment. To do this, we performed Western blot to measure the levels of autophagy markers LC3B and P62 after TBC1D15 knockdown. The results indicate that TBC1D15 knockdown significantly increases the LC3BII levels in microglia after Aβ treatment while decreasing P62 levels (Fig. 8A-8C). Additionally, it leads to a reduction in galectin3 and TREM2 levels (Fig. 8D-8G). Moreover, TBC1D15 knockdown also reduces the lysosomal volume in Aβ-treated BV2 cells (Fig. 8H, 8I), increases ATP levels within the lysosomes (Fig. 8J, 8K), and lowers the pH (Fig. 8L, 8M). Interestingly, TBC1D15 knockdown enhances Aβ phagocytosis (Fig. 8N, 8O), despite a reduction in TREM2 levels (Fig. 8D, 8G). These findings suggest that knocking down TBC1D15 alleviates the autophagy impairment in microglial cells caused by Aβ, improves lysosomal integrity and internal environment, and promotes Aβ phagocytosis. A decrease in autophagy in microglia from 12-month-old 3xTg AD mice was observed, accompanied by an increase in TBC1D15 levels in these cells, suggesting that TBC1D15 elevation may be regulated by autophagy. To investigate whether Aβ oligomers contribute to the upregulation of TBC1D15 in microglia by inhibiting autophagy, rapamycin was used to activate this process. It was found that TBC1D15 levels in BV2 cells treated with both Aβ oligomers and rapamycin were significantly lower than in cells treated with Aβ oligomers alone, which was also associated with a significant decrease in galectin3 expression. This indicates that Aβ oligomers may upregulate TBC1D15 in microglia through autophagy inhibition. To further elucidate whether the reduction of TBC1D15 occurs also via the proteasomal pathway, the proteasome inhibitor MG-132 was administered alongside Aβ oligomers and rapamycin (Fig. 8P-8R). The results revealed an increase in TBC1D15 expression levels. These findings suggest that Aβ oligomers may elevate TBC1D15 expression through the inhibition of both autophagy and the proteasomal pathway.

### Knockdown of TBC1D15 enhances lysophagy and reduces plaque number in the brain tissue of 3xTg AD mice

**Figure 9.**
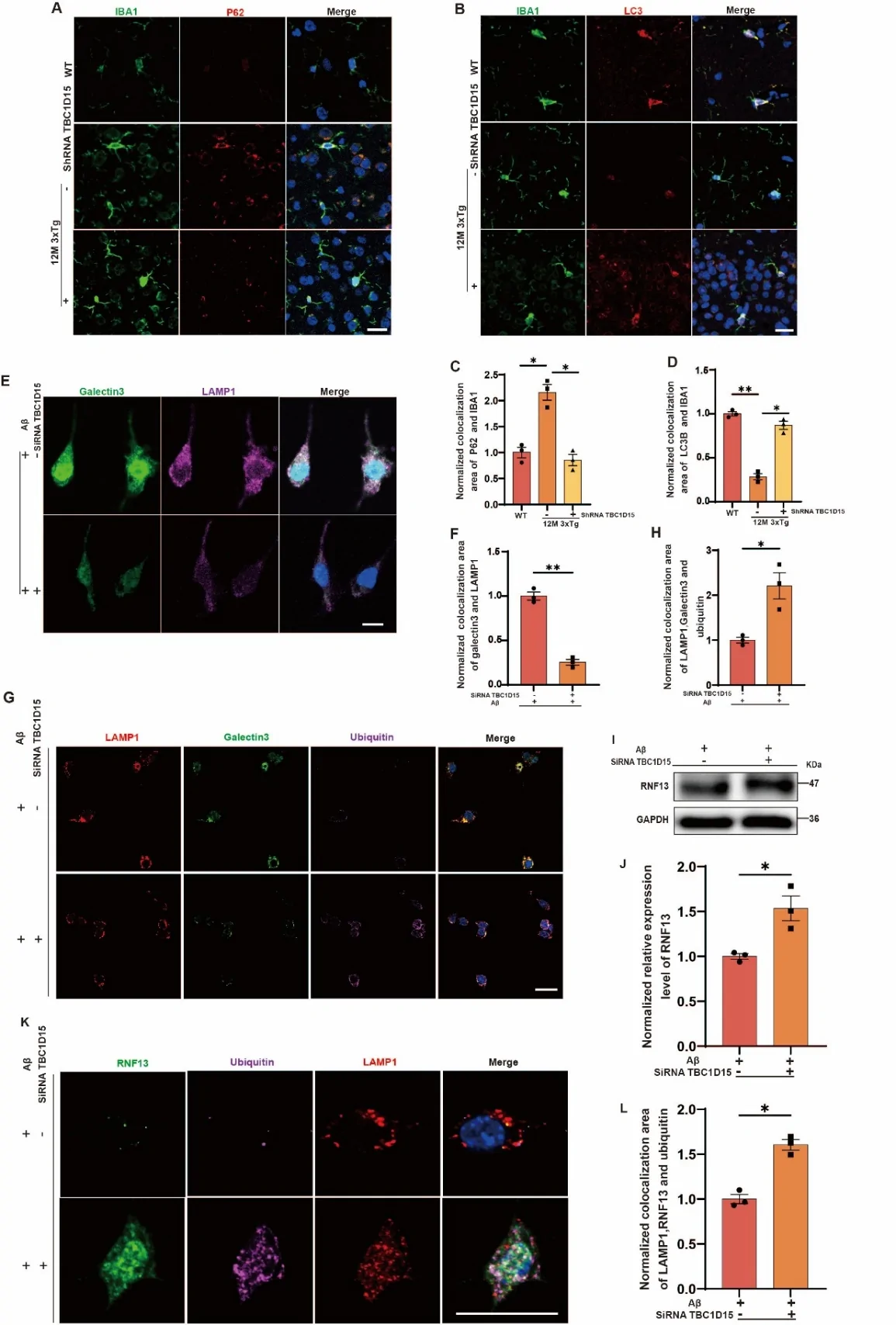
**Knockdown of TBC1D15 can promote autophagy in microglial cells by enhancing lysophagy in 3xTg AD mice**. (A, B) Sections from wild-type and 12-month-old 3xTg AD mice, as well as 3xTg AD mice expressing TBC1D15 shRNA virus driven by the IBA1 promoter, were incubated with antibodies against IBA1 (green), LC3B (red), and P62 (red). n=3/group. Scale bars: 25 μm. (C, D) A comparative quantitative analysis was conducted to determine the area size of P62 and LC3B-positive particles in IBA1-labeled cells. n=3/group. (**E**) BV2 cells were treated with Aβ oligomers or Aβ oligomers combined with siRNA TBC1D15. The levels of galectin3 (green) and LAMP1 (purple) were observed using immunofluorescence. n=3/group. Scale bars: 10 μm. (**F**) A relative quantitative analysis was conducted to assess the colocalization levels of galectin3 and LAMP1 proteins. n=3/group. (**G**) BV2 cells were treated with Aβ oligomers or Aβ oligomers combined with siRNA TBC1D15, and immunofluorescence was used to observe the levels of ubiquitin protein (purple), galectin3 protein(green) LAMP1 protein (red). n=3/group. Scale bars: 20 μm. (**H**) A relative quantitative analysis was performed to assess the colocalization levels of ubiquitin, galectin3, and LAMP1.n=3/group. (**I**) BV2 was treated with siRNA control or siRNA TBC1D15. After 24h transfection, all of them were treated with Aβ for 12h. After that, Western blot was performed to detect RNF13. n=3/group. (**J**) The analysis of normalized relative expression of RNF13. n=3/group. (**K**) BV2 was treated with siRNA control or siRNA TBC1D15. After 24h transfection, all of them were treated with Aβ for 12h. After that, immunofluorescence was performed to detect the colocalization area of RNF13, LAMP1, and ubiquitin. n=3/group, scale bar=20 µm. (**L**) The analysis of normalized colocalization area of LAMP1, RNF13, and ubiquitin. n=3/group. Data from all experiments are expressed as mean ± standard error of the mean (SEM). Non-parametric tests were used for data that did not follow a normal distribution. Nonparametric tests for two independent samples were performed with the Wilcoxon rank-sum test. For nonparametric tests between multiple groups, the Kruskal-Wallis test followed by Dunn’s multiple comparison test was used. Significance levels are indicated by p-values < 0.05(*), and p < 0.01(**).

**Figure 10.**
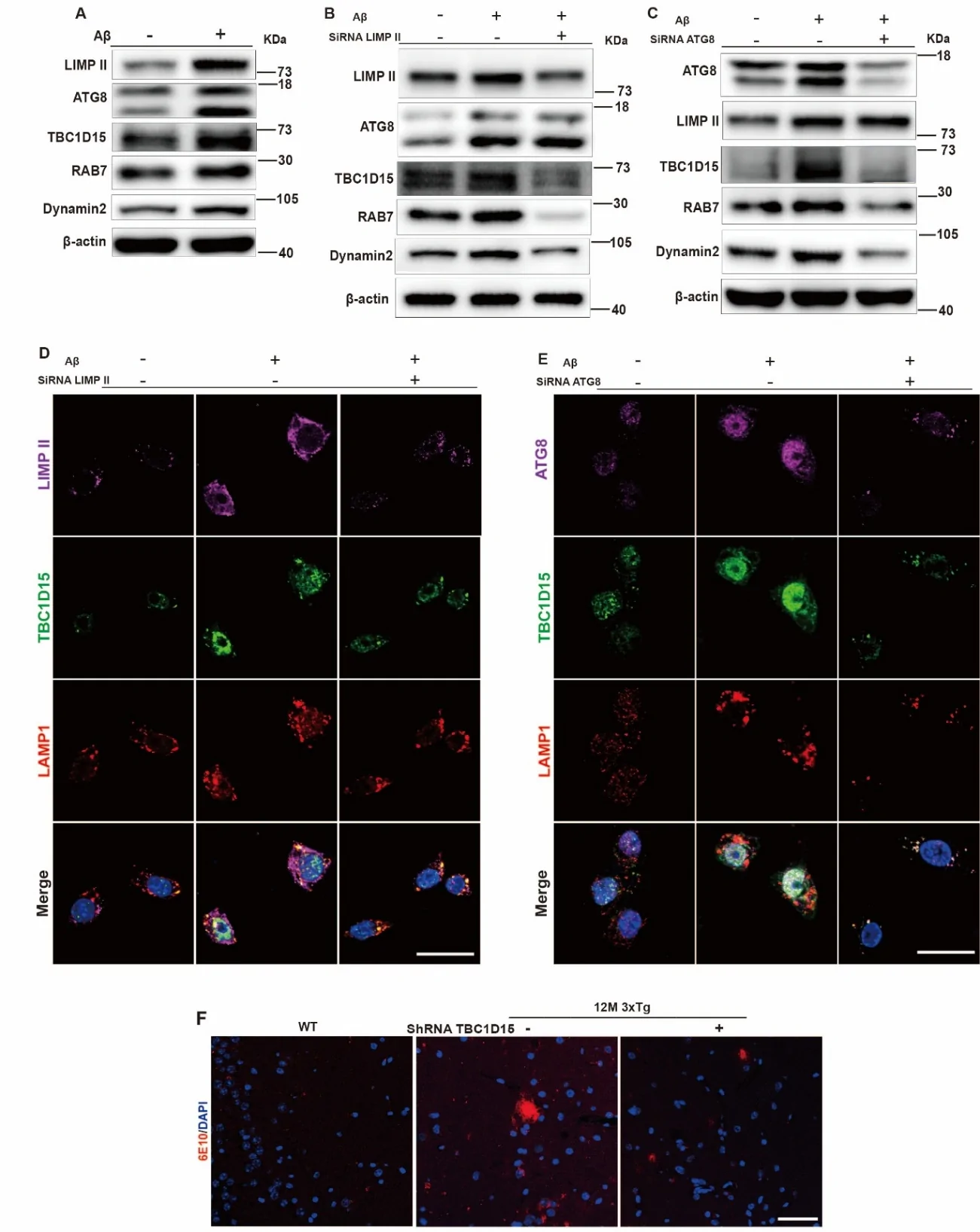
**LIMP II/ATG8-TBC1D15-Dynamin2/RAB7 pathway might involve microglia lysosome membrane swelling in AD**. (**A**) BV2 was treated with Aβ and controlled with PBS, and the Western blot showed the expression level of LIMP II, ATG8, TBC1D15, Dynamin2, and RAB7. n=3/group. (**B**) BV2 was treated with Aβ or Aβ combined with siRNA LIMP II, controlled with PBS. Western blot showed the expression level of LIMP II, ATG8, TBC1D15, Dynamin2, and RAB7. n=3/group. (**C**) BV2 was treated with Aβ or Aβ combined with siRNA ATG8 and controlled with PBS. Western blot showed the expression level of LIMP II, ATG8, TBC1D15, Dynamin2, and RAB7.n=3/group. (**D**) BV2 was treated with Aβ or Aβ combined with siRNA LIMP II, controlled with PBS. Immunofluorescence showed the co-localization of LAMP1, LIMP II, and TBC1D15.n=3/group. Scale bar=20μm. (**E**) BV2 was treated with Aβ or Aβ combined with siRNA ATG8 and controlled with PBS. Immunofluorescence showed the co-localization of LAMP1, ATG8, and TBC1D15.n=3/group. Scale bar=20 μm. F. Brain slices from these animals were subjected to 6E10 antibody staining (red). n=3/group. Scale bars: 50 μm. (Statistical analysis graphs are available in Figure S3 and Figure S4.)

Since knocking down TBC1D15 can improve autophagic damage caused by Aβ oligomers, and considering the close relationship between lysosomes and autophagy, we investigated whether TBC1D15 knockdown can improve lysosomal damage. Knocking down TBC1D15 significantly alleviates lysosomal damage, particularly by reducing galectin3 levels in the lysosomes of BV2 cells (Fig. 9E, 9F). Further analysis revealed that TBC1D15 knockdown increased LC3 levels and decreased P62 levels in the brain tissues of 3xTg AD mice (Fig. 9A, 9B, 9C, 9D). Besides, we found that TBC1D15 knockdown in microglia reduces amyloid plaque accumulation in 3xTg AD mice (Fig. 10F, Supplementary Fig. 4E). This indicates that TBC1D15 enhances autophagy in the microglia of 3xTg AD mice, likely by promoting lysosomal autophagy. This raises the question: what mechanism allows TBC1D15 knockdown to ameliorate lysosomal damage? Lysophagy plays a crucial role in removing damaged lysosomes and preventing their accumulation [42]. To investigate how TBC1D15 knockdown improves microglial lysosomal integrity through enhancing lysophagy, after treatment, we evaluated the ubiquitination level of lysosome of lysosomal membrane protein LAMP1 in the damaged lysosome of Aβ-treated microglia using immune-fluorescence (Fig. 9G). The results showed that TBC1D15 knockdown significantly increased the ubiquitination of LAMP1 in microglia after Aβ oligomer treatment (Fig. 9H). These suggest that TBC1D15 knockdown can increase lysophagy to the damaged lysosome.

By what mechanism does knockdown TBC1D15 increase lysophagy? Since RNF13 is an E3 ligase for LAMP1 ubiquitination [43], we investigate whether TBC1D15 knockdown promotes LAMP1 ubiquitination through RNF13. BV2 was transfected with siRNA control or siRNA TBC1D15 for 24 h and treated with Aβ for 12 h. After that, RNF13 expression level was detected by western blot, and it was found that the level of RNF13 increased after knocking down TBC1D15 (Fig. 9I, 9J). The immunofluorescence results showed that the co-localization area of ubiquitin, LAMP1, and RNF13 increased after the knockdown of TBC1D15(Fig. 9K, 9O). These suggest that knocking down TBC1D15 may increase LAMP1 ubiquitin levels by increasing the RNF13 level. Taken together, these findings further suggest that TBC1D15 knockdown in microglia may restore autophagy and enhance lysosomal autophagy to clear Aβ, thereby reducing amyloid plaque formation in 3xTg AD mice.

### LIMP II/ATG8-TBC1D15-Dynamin2/RAB7 involved microglia lysosome membrane swelling in AD

A type II lysosomal integral membrane protein (LIMP II) and ATG8 (LC3/GABARAP proteins in mammals) can recruit TBC1D15 to the damaged lysosomal membrane [16]. To clarify whether LIMP II and ATG8 are involved in regulating the role of TBC1D15 in lysosomal swelling, BV2 cells were treated with Aβ oligomers and the results showed that LIMP II, ATG8, and TBC1D15 increased by western blot (Fig. 10A, Supplementary Fig. 3B, S3C, S3E). This suggests that TBC1D15 may be regulated by LIMP II and ATG8. To further clarify whether LIMP II and ATG8 are involved in the regulation of TBC1D15 to participate in lysosomal swelling, BV2 cells were treated with siRNA LIMP II and siRNA ATG8. Aβ combined with siRNA LIMP II could decrease TBC1D15 levels compared with Aβ alone (Fig. 10B, Supplementary Fig. 3G, 3H). Immunofluorescence showed TBC1D15 has less colocalization with LAMP1 in BV2 treated with siRNA LIMP II than siRNA control (Fig. 10D, Supplementary Fig. 4A, 4B). Aβ combined with siRNA ATG8 could decrease TBC1D15 levels compared with Aβ alone (Fig. 10C, Supplementary Fig. 3M, 3O). Immunofluorescence showed TBC1D15 has less colocalization with LAMP1 in BV2 treated with siRNA ATG8 than siRNA control (Fig. 10E, Supplementary Fig. 4C, 3D). Meanwhile, we found that LIMP II knockdown could not regulate ATG8 level and ATG8 knockdown also could not regulate LIMP II level (Fig. 10B, 10C, Supplementary Fig. 3J, L). These indicate that LIMP II and ATG8 regulate TBC1D15 respectively after Aβ oligomers treatment.

How does TBC1D15 regulate swelling lysosomes in AD? Dynamin2 is a large GTPase that regulates vesicle trafficking. Dynamin2 can facilitate lysosome reformation and RAB7 can also promote lysosomal membrane regeneration [44-46]. They are both molecules downstream of TBC1D15 that promote membrane damage repair [16]. To investigate how dose TBC1D15 regulates swelling lysosomes in AD. BV2 cells were treated with Aβ oligomers, and results showed that the levels of dynamin2 and RAB7 increased by western blot (Fig. 10A, Supplementary Fig. 4A, 4D), indicating that TBC1D15 regulates the swelling lysosomes by increasing the levels of dynamin2 and RAB7. Meanwhile, we found that when knockdown LIMP II or ATG8 reduced TBC1D15 levels, dynamin2, and RAB7 levels decreased (Fig. 10B, 10C, Supplementary Fig. 3F, 3I, 3K, 3N). These suggest that the LIMP II/ATG8-TBC1D15-Dynamin2/RAB7 may participate in maintaining microglia lysosome membrane swelling in AD.

## DISCUSSION

Microglia in AD brain can process and clear phagocytosed Aβ aggregates [47, 48], hyper-phosphorylated tau aggregates [49, 50], as well as inflammatory pathways mediators through autophagy [51-53]. Therefore, the autophagic capacity of microglia plays a crucial role in inhibiting the pathological progression of AD. However, as AD progresses, the autophagic ability of microglia declines [54, 55], leading to a reduced capacity to process and resolve pathological molecules. Numerous studies have shown that enhancing microglial autophagy can significantly improve AD pathology and cognitive dysfunction [5, 54]. Thus, improving autophagic dysfunction in microglia is an important therapeutic target for addressing AD pathology. However, the molecular mechanisms underlying microglial autophagic dysfunction in AD remain unclear.

Despite much research focused on autophagic dysfunction, the key mechanism remains unclear. Current research shows that the levels of autophagy in microglia of 3-month, 6-month, 9-month, and 12-month-old 3xTg AD mice exhibit a dynamic change from enhancement to reduction. Our research will provide precise temporal and cell-type references for further studies on autophagy related to AD at different stages in the future. What are the key mechanisms behind the dynamic changes of autophagy? It was found that these dynamic changes in autophagy are accompanied by progressively exacerbated lysosomal swelling damage. Since lysosomes are the executors of autophagy [56], damaged lysosomes are unable to degrade the Aβ contents of autophagosomes, leading to the inhibition of autophagy through feedback mechanisms [57].

By comparing the relationship between lysosomal damage and autophagy in BV2 cells, C8D1A cells, and HT22 cells, it was discovered that only in BV2 cells, lysosomal damage and deterioration of the lysosomal internal environment emerged, particularly characterized by decreased ATP levels and increased pH within the lysosomes and a decline in autophagy occurred in a time- and concentration-dependent manner following the phagocytosis of Aβ oligomers (Fig. 4, 5, Supplemental Fig. 1). In contrast, the latter two cell types did not exhibit lysosomal damage and a decline in autophagy after the phagocytosis of Aβ oligomers. These indicate that lysosomal damage is involved in the decline of autophagy. It also further suggests that lysosomal damage of microglia in 3xTg AD mice is involved in the dynamic changes of autophagy in microglia. These changes primarily occur in microglia rather than in astrocytes or neurons, as we observed these changes only in BV2 cells and not in C8D1A or HT22 cells. The difference may be generated by the different engulfing Aβ. Microglia included Aβ mainly through receptor-mediated phagocytosis and the other cells through endocytosis [58]. Endocytosis can reach a saturation point after internalizing certain substances, inhibiting further uptake of those substances. Receptor-dependent phagocytosis, on the other hand, may experience sustained capacity without reaching saturation due to an increase in the phagocytic receptors. The endocytosis of Aβ oligomers stimulates the expression of the Aβ phagocytic receptor TREM2 [59, 60], resulting in a positive feedback loop for the phagocytosis. The effects of Aβ oligomers on lysosomes and autophagy in microglia may also depend on this enhanced phagocytic feedback.

The swelling of microglial lysosomes plays a key role in the triggering and development of neuroinflammation [61]. Previous studies also showed the inhibition of microglia can improve cognitive dysfunction in AD [62, 63]. However, microglia also played an important role in normal cognition [64, 65]; the deletion of microglia may contribute to other side effects despite improving cognitive dysfunction in AD [66, 67]. So, it was not a good means for microglia deletion to treat AD. Therefore, it is necessary to develop a method that can regulate the function of microglia to modulate the neuroinflammation caused by microglia in AD or to facilitate the clearance of Aβ.

Present study showed that in the microglia of 12-month-old mice, the expression level of TBC1D15, as the lysosomal repair-related molecule, was significantly elevated. TBC1D15 is involved in the repair of lysosomal membrane damage [15]. When the lysosome was damaged, TBC1D15 was recruited to damaged lysosome membrane to initiate the membrane repairing [16]. Therefore, in microglia treated with Aβ oligomers or in microglia from 3xTg AD mice, the increased expression levels of lysosomal TBC1D15 are likely aimed at repairing damaged lysosomes. TBC1D15 could participate in lysosomal enlargement of volume induced by Aβ. TBC1D15 can bind to ATG8 on the damaged lysosomal membrane, recruiting other molecules to repair the lysosomal membrane. However, this repair is incomplete [16]. Is it possible that the damaged lysosomes are delayed in entering autophagy for elimination? Besides, TBC1D15 is a RAB7 GTPase that can hydrolyze GTP to generate GDP [13, 68], thereby inhibiting the activity of Rab. RAB7 plays an important role in lysosome biogenesis [69-71].

Downregulation of TBC1D5 can activate RAB7 and promote lysosome formation [18]. However, the knockdown of TBC1D15 unexpectedly resulted in no significant increase in lysosome number and could inhibit the increase in lysosomal volume and Ph value increasing after Aβ treatment and galectin3 level, a biomarker of lysosomal damage, increasing in the present study. Moreover, our study found that in Aβ-treated BV2 cells, there was a decrease in lysosomal ubiquitination, and knockdown of TBC1D15 was able to restore lysophagy. Besides, TBC1D15 hydrolysize GTP to make RAB7 change into an inactive GDP-bound state from an active GTP-bound state [21, 46]. But RAB7 can foster damaged lysosome into lysophagy for degrading the the damaged lysosme [72-74]. Therefore, the knockdown of TBC1D15 may promote lysophagy by activating RAB7, thereby clearing damaged lysosomes, maintaining the overall membrane integrity and normal function of lysosomes, and enhancing the autophagic function of microglia, which in turn enables microglia to effectively clear Aβ. Due to the knockdown of TBC1D15 reducing TREM2 and increasing the phagocytosis of Aβ, the knockdown of TBC1D15 promotes Aβ phagocytosis by enhancing autophagy; however, the receptors responsible for the enhanced phagocytosis need further identification. Other studies have shown that TBC1D15 can promote mitochondrial DNA damage and the generation of cytosolic DNA-dependent protein kinase catalytic subunit (DNA-PKcs), which may subsequently activate the cGAS-STING pathway to enhance LCN2 expression and inhibit autophagy [75]. TBC1D15 may also suppress autophagy by inhibiting the formation of autophagosomes through its regulation of RAB7 [75]. Additionally, TBC1D15 may inhibit mitophagy by disrupting the interaction between mitochondria and lysosomes, thereby reducing energy production and suppressing the energy-consuming process of autophagy [18, 21]. These mechanisms may also play a role in the impaired autophagy of microglia in AD. Further investigation into these mechanisms is needed in future studies.

**Figure 11.**
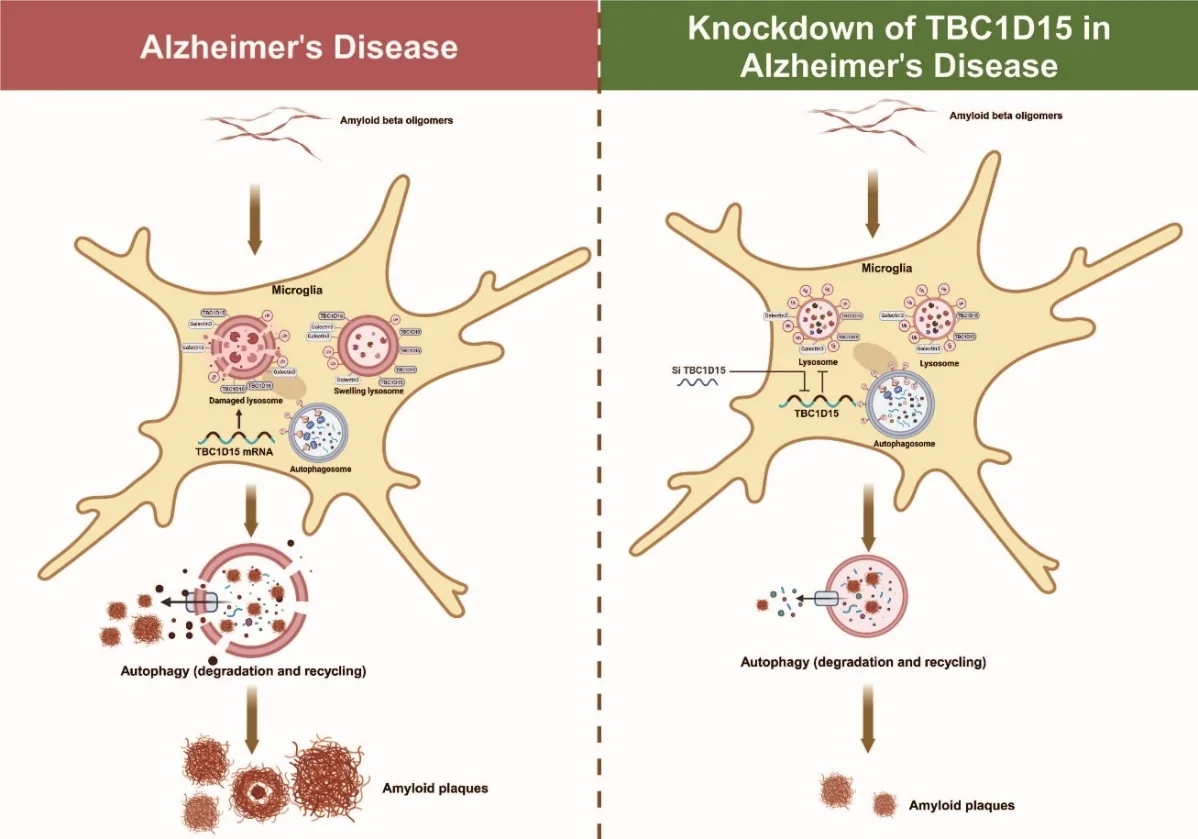
**Schematic diagram of the mechanism of this study**. (Created with BioRender.com)

This study, based on a mouse AD model and a mouse microglial cell line, found that TBC1D15 knockdown could improve microglial autophagy and alleviate AD pathology. However, in recent years, molecular mechanisms and drug development findings based on mouse models of AD or other neurodegenerative diseases have not shown corresponding effects in primates or humans [76-78]. This is largely due to species differences. Therefore, whether knockdown of TBC1D15 can truly improve microglial autophagy in the brain tissue of AD patients requires further validation in non-human primate models. Microglial autophagy plays an essential role in the development and plasticity of normal neuronal synaptic structures [79-83]. However, during external disturbances such as inflammation, it becomes excessively activated, leading to over-autophagy of synaptic proteins and resulting in synaptic damage [84-86]. In the later stages of AD, microglial autophagy declines, and the ability to phagocytose and clear amyloid-beta (Aβ) is impaired, leading to Aβ accumulation [51, 54, 87]. Currently, knockdown of TBC1D15 has been shown to improve microglial autophagy and promote Aβ clearance. However, whether sustained enhancement of microglial autophagy through TBC1D15 inhibition could have adverse effects on neuronal synaptic structures in the brain requires further investigation in future studies. This will help clarify whether suppression of TBC1D15 could pose potential harmful side effects on brain health and provide a basis for the development of AD therapeutics targeting TBC1D15 in the future.

Current research has found that downregulating TBC1D15 can significantly improve lysosomal swelling, reduce microglial phagocytosis of Aβ, enhancing lysophagy and reduce the formation of amyloid plaques in AD [16]. These results suggest that TBC1D15 may potentially serve as a potentially important biomarker for AD diagnosis and therapeutic target. In future studies, the protein structures of TBC1D15 (both secondary and tertiary) will be predicted using AlphaFold, to screen and design small molecule inhibitors targeting TBC1D15, as well as drugs that inhibit its expression or promote its degradation [88, 89]. These drugs will be modified to enable effective delivery across the blood-brain barrier (BBB) to brain tissue, and to specifically enter microglial cells to inhibit TBC1D15 in microglia. This approach could potentially provide effectively therapeutic agents for AD. Additionally, during the development of these drugs, the effects of different dosages on inhibiting microglial autophagy and reducing Aβ plaques in AD brain tissue will be closely monitored to avoid damage to brain tissue caused by excessive activation of microglial autophagy. The pharmacokinetics of these drugs will be measured and observed, with particular attention paid to drug administration timing, intervals, and frequency, in order to maintain optimal blood drug concentrations and ultimately achieve therapeutic efficacy for AD.

## Supplementary Materials

The Supplementary data can be found online at: www.aginganddisease.org/EN/10.14336/AD.2024.1373.
