# Supplementary material for: TBC1D15 Inhibits Autophagy of Microglia through Maintaining the Damaged Swelling Lysosome in Alzheimer’s Disease
Source: Aging Dis. 2024 Dec 13;16(6):3601–24. doi: 10.14336/AD.2024.1373 (PMC12539536; doi:10.14336/AD.2024.1373)
Supplement: Supplementary file 1 — The Supplementary data can be found online at: www.aginganddisease.org/EN/10.14336/AD.2024.1373. [file AD-16-6-3601-s.pdf]

SUPPLEMENTARY DATA

TBC1D15 Inhibits Autophagy of Microglia through Maintaining the Damaged Swelling Lysosome in Alzheimer's Disease

You Wu, Yong-ming Zhou, Wei Wu, Wan-rong Jiang, Xin-yuan Zhang, Si-yuan Song, Zhao-hui Yao

© 2024. Wu Y. et al. Published online at http://www.aginganddisease.org/EN/10.14336/AD.2024.1373

SUPPLEMENTARY DATA

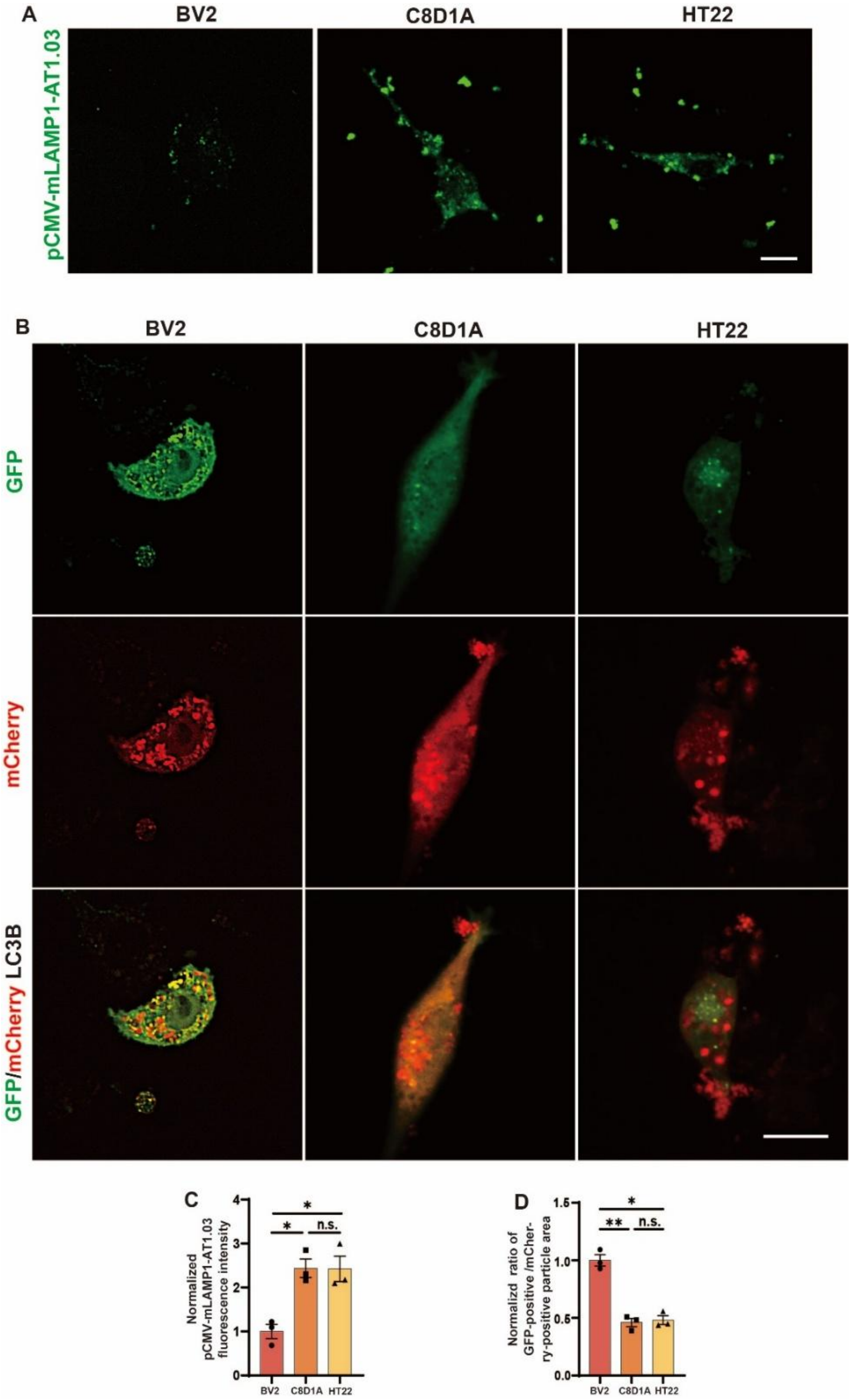

Supplementary Figure 1. There was no significant change in lysosomal pH values and lysosomal ATP levels in HT-22 and C8D1A cells. A.BV2 cells, C8D1A, and HT22 cells were incubated with 10 μM Aβ, the fluorescence intensity of AT1.03-LAMP1 protein was normalized for analysis. n=3/group. Scale bars: 10 μm. B. BV2 cells, C8D1A, and HT22 cells were incubated with 10 μM Aβ, the GFP-mCherry-LC3 protein was employed to assess the pH levels inside lysosomes. n=3/group. Scale bars: 10 μm. C. After Aβ oligomer treatment of BV2, C8D1A and HT-22 cells 24 hours, the fluorescence intensity of AT1.03-LAMP1 protein was normalized for analysis. n=3/group. D. After Aβ oligomer treatment of BV2, C8D1A and HT-22 cells 24 hours, the ratio of GFP-positive particle area to mCherry-positive particle area was normalized to assess lysosomal pH.n=3/group. Data from all experiments are expressed as mean ± standard error of the mean (SEM). Nonparametric tests were used for data that did not follow a normal distribution. Nonparametric tests for two independent samples were performed with the Wilcoxon rank-sum test. For nonparametric tests between multiple groups, the Kruskal-Wallis test followed by Dunn's multiple comparison test was used. Significance levels are indicated by p-values < 0.05(*), p < 0.01(**).

© 2024. Wu Y. et al. Published online at http://www.aginganddisease.org/EN/10.14336/AD.2024.1373

SUPPLEMENTARY DATA

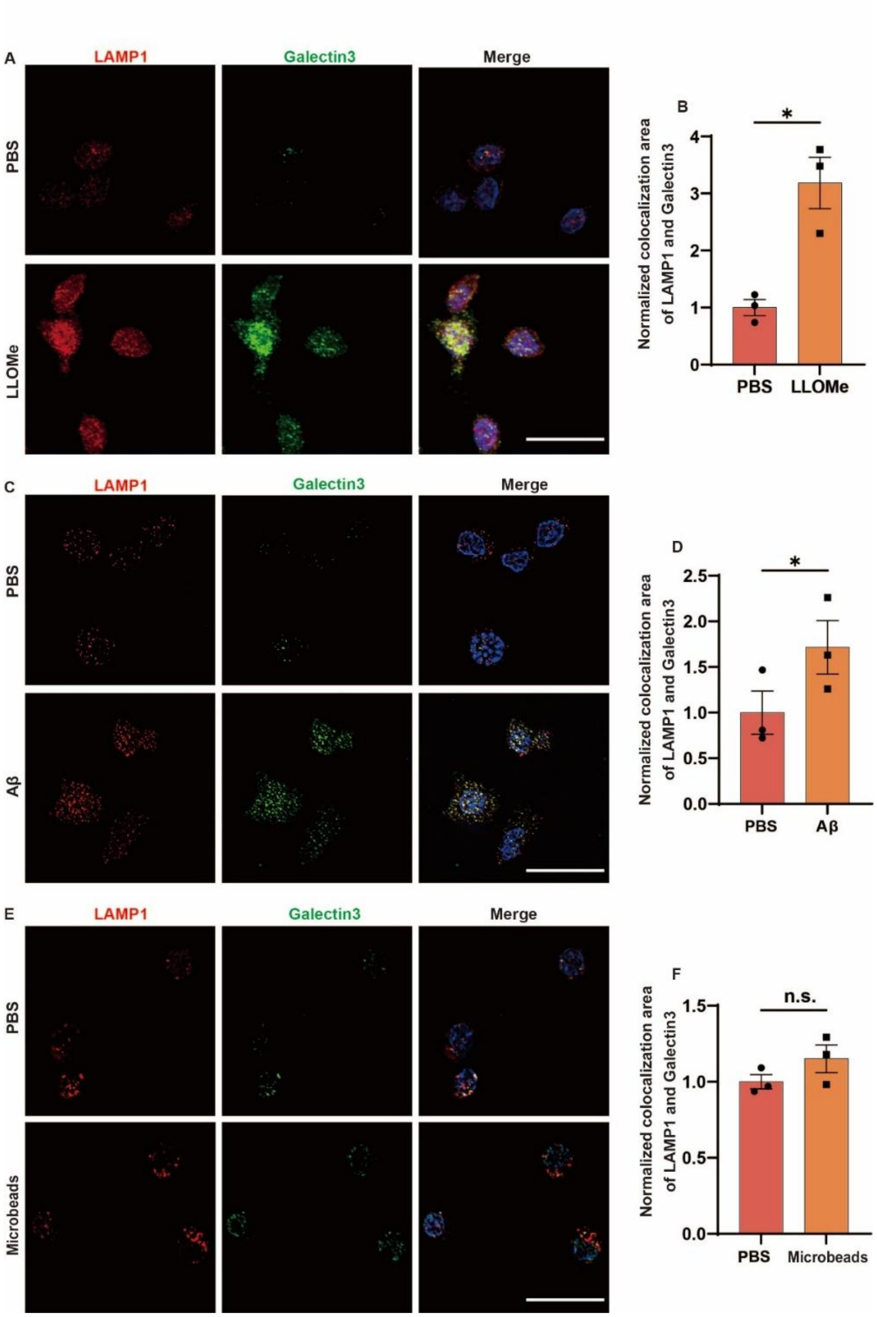

Supplementary Figure 2. Lysosome damage increased after LLOMe, Aβ treatment, but no significant lysosome damage was observed after microbeads treatment. A. BV2 cells were treated with LLoMe and the control group was treated with PBS for immunofluorescence of galectin3 and LAMP1. n=3/group, scale bar=20 μm.B. The analysis of normalized colocalization area of LAMP1 and galectin3.n=3/group. C. BV2 cells were treated with Aβ and the control group was treated with PBS for immunofluorescence of galectin3 and LAMP1. n=3/group, scale bar=20 μm. D. The analysis of normalized colocalization area of LAMP1 and galectin3.n=3/group. E. BV2 cells were treated with microbeads and the control group was treated with PBS for immunofluorescence of galectin3 and LAMP1. n=3/group, scale bar=20 μm. F. The analysis of normalized colocalization area of LAMP1 and galectin3.n=3/group. Data from all experiments are expressed as mean ± standard error of the mean (SEM). Non-parametric tests were used for data that did not follow a normal distribution. Nonparametric tests for two independent samples were performed with the Wilcoxon rank-sum test. For nonparametric tests between multiple groups, the Kruskal-Wallis test followed by Dunn's multiple comparison test was used. Significance levels are indicated by p-values < 0.05(*).

© 2024. Wu Y. et al. Published online at http://www.aginganddisease.org/EN/10.14336/AD.2024.1373

SUPPLEMENTARY DATA

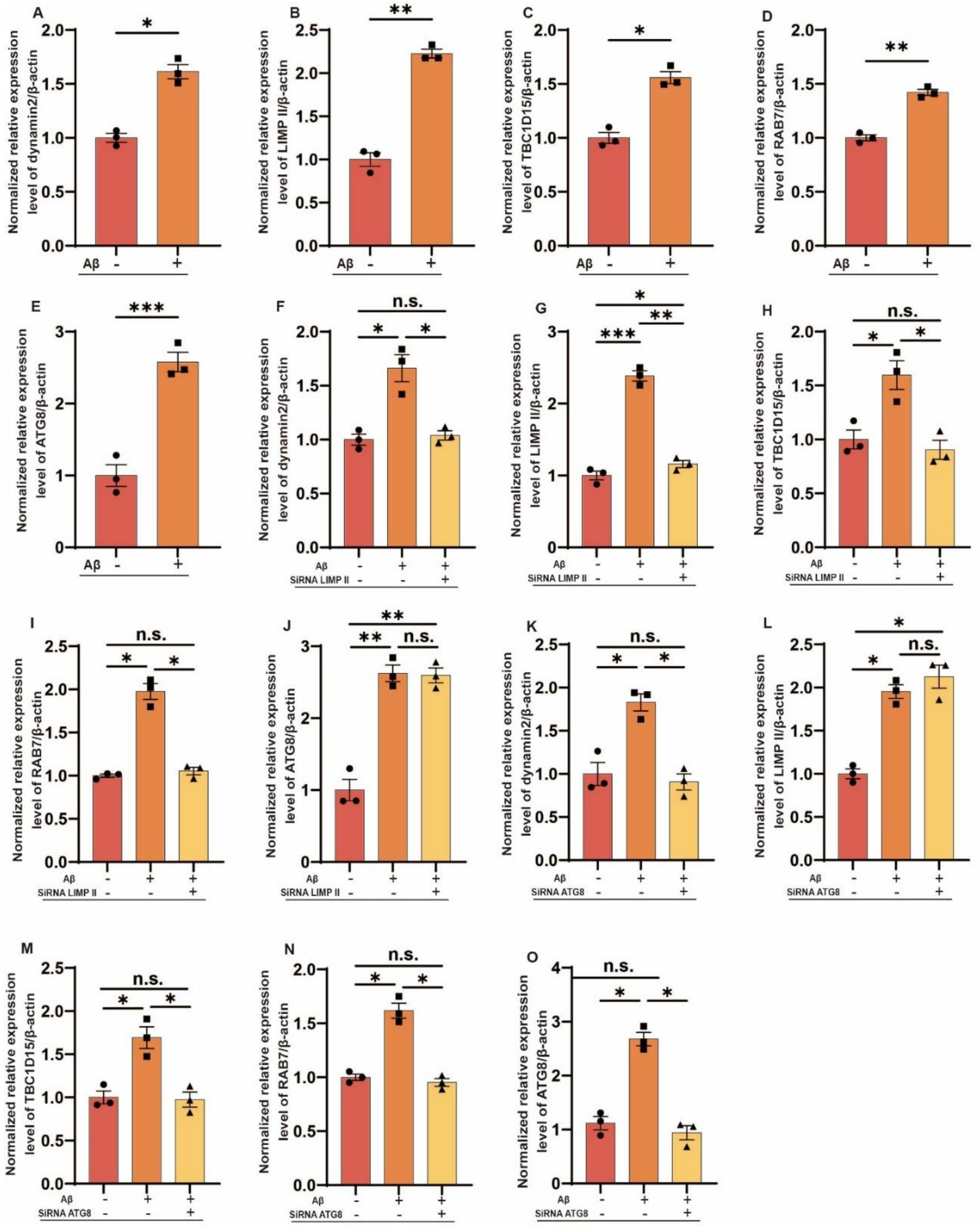

Supplementary Figure 3. Statistical analysis of western blot in Figure 10. A-E. Western blot analysis of normalized Dynamin2, LIMP II, TBC1D15, RAB7 and ATG8 protein expression levels in BV2 cells after the treatment of Aβ and PBS. n=3/group. F-J. Western blot analysis of normalized Dynamin2, LIMP II, TBC1D15, RAB7 and ATG8 protein expression levels in BV2 cells. n=3/group. K-O. Western blot analysis of normalized Dynamin2, LIMP II, TBC1D15, RAB7 and ATG8 protein expression levels in BV2 cells. n=3/group. Data from all experiments are expressed as mean ± standard error of the mean (SEM). Non-parametric tests were used for data that did not follow a normal distribution. Nonparametric tests for two independent samples were performed with the Wilcoxon rank-sum test. For nonparametric tests between multiple groups, the Kruskal-Wallis test followed by Dunn's multiple comparison test was used. Significance levels are indicated by p-values < 0.05(*), p < 0.01(**), p < 0.001(***).

© 2024. Wu Y. et al. Published online at http://www.aginganddisease.org/EN/10.14336/AD.2024.1373

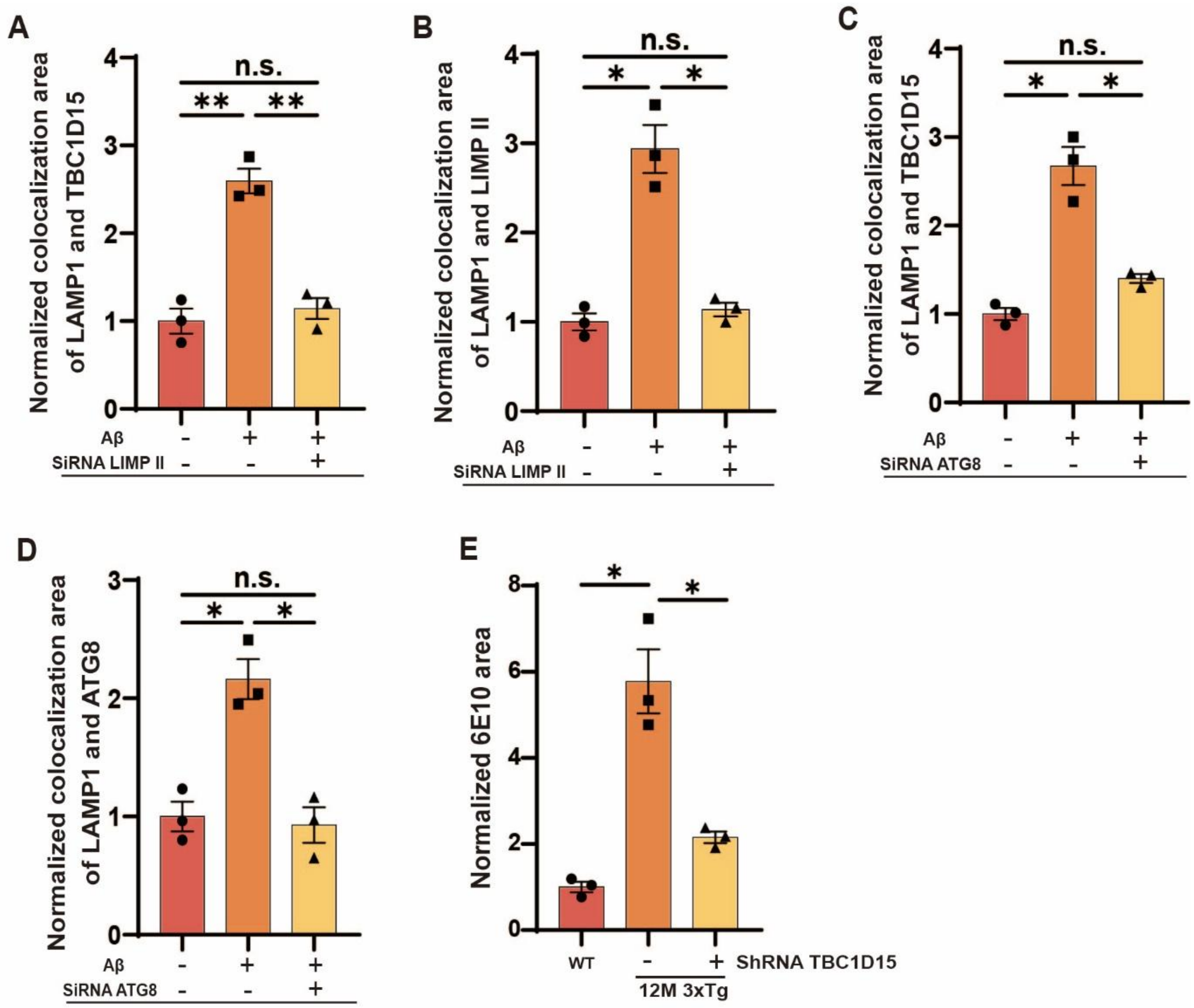

Supplementary Figure 4. Statistical analysis of immunofluorescence in Figure 10. A. Normalized analysis of colocalization area of LAMP1 and ATG8. N=3/group. B. Normalized analysis of colocalization area of LAMP1 and LIMP II. n=3/group. C. Normalized analysis of colocalization area of LAMP1 and TBC1D15. n=3/group. D. Normalized analysis of colocalization area of LAMP1 and TBC1D15. n=3/group. E. The statistical analysis was performed on the 6E10-positive plaques. n=3/group. Data from all experiments are expressed as mean ± standard error of the mean (SEM). Non-parametric tests were used for data that did not follow a normal distribution. Nonparametric tests for two independent samples were performed with the Wilcoxon rank-sum test. For nonparametric tests between multiple groups, the Kruskal-Wallis test followed by Dunn's multiple comparison test was used. Significance levels are indicated by p-values < 0.05(*), p < 0.01(**), p <
0.001(***).

© 2024. Wu Y. et al. Published online at http://www.aginganddisease.org/EN/10.14336/AD.2024.1373
